# Multiparametric MRI-based biomarkers in the non-fluent and semantic variants of primary progressive aphasia

**DOI:** 10.1007/s00415-025-13215-9

**Published:** 2025-07-02

**Authors:** Marco Michelutti, Hans-Jürgen Huppertz, Heiko Volkmann, Sarah Anderl-Straub, Daniele Urso, Benedetta Tafuri, Salvatore Nigro, Paolo Manganotti, Leonie Werner, Jolina Lombardi, Markus Otto, Giancarlo Logroscino, Hans-Peter Müller, Jan Kassubek

**Affiliations:** 1 Neurology Unit, Department of Medical, Surgical and Health Sciences, University of Trieste, Strada di Fiume 447, 34149 Trieste, Italy; 2 Department of Neurology, University Hospital Ulm, Ulm, Germany; 3 Center for Neurodegenerative Diseases and the Aging Brain, University of Bari Aldo Moro at Pia Fondazione “Card. G. Panico”, Tricase, Italy; 4 Swiss Epilepsy Clinic, Klinik Lengg, Zurich, Switzerland; 5 Department of Engineering of Innovation, University of Salento, Lecce, Italy; 6 Institute of Nanotechnology, National Research Council (CNR-NANOTEC) c/o Campus Ecotekne, via Monteroni, 73100 Lecce, Italy; 7 Department of Neurology, University Hospital Halle, Martin Luther University, Halle (Saale), Germany; 8 Department of Diagnostic and Interventional Radiology, University Hospital Ulm, Ulm, Germany; 9 Department of Nuclear Medicine, University Hospital Ulm, Ulm, Germany

**Keywords:** Primary progressive aphasia, Diffusion tensor imaging, Longitudinal, Fractional anisotropy

## Abstract

**Background:**

The non-fluent (nfPPA) and semantic (svPPA) variants of primary progressive aphasia exhibit distinct clinical features. We investigated whether diffusion tensor imaging (DTI) and atlas-based volumetry (ABV) could reveal divergent patterns of longitudinal changes in brain white matter microstructure and gray matter volumes.

**Methods:**

MRI datasets from 29 nfPPA, 27 svPPA, and 39 controls were analyzed. White matter fractional anisotropy (FA) differences were assessed using unbiased Whole Brain-based Spatial Statistics (WBSS) and Tract-Wise Fractional Anisotropy Statistics (TFAS). Gray matter volumetric differences were calculated by Atlas-Based Volumetry (ABV). A subset of 10 nfPPA and 6 svPPA patients underwent longitudinal MRI at 12 months. FA maps were correlated with disease severity (FTLD-CDR sum of boxes). A random forest classifier validated tracts of interest (TOI) and structures of interest (SOIs) selection as a proof-of-concept.

**Results:**

At baseline, nfPPA showed frontal, callosal, and temporal white matter degeneration, while the left inferior longitudinal fasciculus (ILF) was predominantly involved in svPPA. Longitudinally, nfPPA exhibited frontal, callosal, and posterior temporal progression, while svPPA showed localized antero-posterior ILF progression. ABV aligned with the DTI analyses, demonstrating volumetric reductions in the frontal lobe for nfPPA and in temporal lobe and subcortical limbic structures in svPPA. Sub-clusters of white matter damage progression correlated with worsening FTLD-CDR scores. Random forest analysis identified the most discriminative TOIs and SOIs.

**Conclusions:**

Distinct degeneration patterns emerged across nfPPA and svPPA, supporting early differential diagnosis and correlating with disease worsening. These findings support the utility of combined DTI and ABV in tracking disease progression.

**Supplementary Information:**

The online version contains supplementary material available at 10.1007/s00415-025-13215-9.

## Introduction

The term frontotemporal lobar degeneration (FTLD) defines a spectrum of conditions that cause degeneration of frontal and temporal lobes and that is associated with a heterogeneous range of age-associated neurodegenerative diseases characterized by changes in behavior, language, executive control, and motor symptoms [1]. The syndromes are currently categorized according to the primary protein components found in intracellular aggregates observed during autopsy, the two primary types of proteinopathies being tauopathies (FTLD-Tau) and TDP-43 (FTLD-TDP43) proteinopathies [2]. From the clinical perspective, these FTLD proteinopathies cause frontotemporal dementia (FTD) [3], the most frequent clinical phenotypes being the behavioral variant of frontotemporal dementia (bvFTD), characterized by changes in socio-emotional function [4], the semantic variant of primary progressive aphasia (svPPA) [5], associated with the loss of knowledge about words and objects, and the non-fluent variant of PPA (nfPPA) [5], marked by articulation difficulties, speech apraxia, and agrammatism. Although the PPA variants are defined by distinct clinical features, substantial overlap between syndromes exists, particularly in early disease stages, and up to 30–40% of patients remain unclassifiable [6, 7] using current consensus criteria [5]; this highlights the diagnostic challenges and underscores the need for in vivo neuroimaging biomarkers to support accurate diagnosis and subtype differentiation. Furthermore, the clinico-pathological correlations between antemortem FTD clinical phenotypes and post-mortem FTLD neuropathological diagnoses are complex [3]. FTD clinical phenotypes associated with FTLD-Tau and FTLD-TDP43 are clinically indistinguishable, and there are currently no diagnostic markers available in vivo to reliably predict the underlying neuropathology [4, 8–10]. Additionally, there is a need to identify and establish sensitive biomarkers in FTLD to facilitate the assessment of progression rates, estimate prognosis, and stratify patients into homogeneous study groups. Translating neuropathological findings into a clinical setting, though challenging, has the potential to enable individualized diagnostic procedures and promote its utilization as a surrogate marker of disease progression to enhance the feasibility of clinical trials [11]. Among the several candidate imaging-based biomarkers for tracking change in FTLD, diffusion tensor imaging (DTI), which measures water diffusion to evaluate microstructural alterations, has been used in both cross-sectional [12–32] and longitudinal studies to assess white [33–40] and gray [26, 38] matter degeneration. Some studies have suggested that white matter abnormalities are an early marker of FTLD as opposed to the volumetric gray matter loss occurring later in the disease [13, 17, 41, 42]. Moreover, longitudinal studies have shown that white matter changes over a 12-month period are more extensive than variations in gray matter [18].

These observations align with the *molecular nexopathy* framework hypothesized to explain the propagation pattern for misfolded proteins in neurodegenerative diseases [43–45]. Both tau [46, 47] and TDP-43 [48] aggregates are thought to spread trans-synaptically following the degree of functional [49, 50] and structural [50] connectivity between regions that belong to the same brain functional networks rather than direct contiguity [51]. This constellation indicates DTI as especially suited for the assessment of the progression of FTLD proteinopathies along white matter fiber bundles, with fractional anisotropy (FA) as the standardized metric [33, 34, 36–39, 52, 53].

The goal of this study was to quantify longitudinal changes in both gray and white matter integrity using a multi-parametric approach to (micro-)structural magnetic resonance imaging (MRI) combining DTI and unbiased volumetry (atlas-based volumetry, ABV) [54–59]. Accordingly, we aimed (1) to identify whole brain-based DTI alteration patterns, white matter Tract-of-Interest (TOI)-guided FA changes, and volumetric differences in gray matter structures of interest (SOIs) for each group; (2) to examine patients with follow-up DTI scans to assess differences from baseline scans, both in an unbiased and a hypothesis-driven manner; and (3) to correlate disease progression scores with TOI-derived DTI data across the entire patient sample.

## Methods

### Subjects and MRI acquisition

Thirty-three patients with a diagnosis of nfPPA and 29 patients with a diagnosis of svPPA were retrospectively included in the study. Controls (*n* = 39) included both 10 healthy subjects and 29 subjects with subjective cognitive decline [60] and negative CSF biomarkers for neurodegeneration (amyloid and tau pathology). For clarity, the term ‘controls’ will henceforth refer collectively to both healthy volunteers and individuals with subjective cognitive decline (SCD) and negative CSF biomarkers.

NfPPA and svPPA were diagnosed according to established diagnostic criteria [5, 61] by specialized board-certified neurologists in all patients. Data acquisition was performed at two study sites (Ulm, Germany and Tricase, Italy). Participants scanned in Ulm are designated as cohort “A”, and those scanned in Tricase as cohort “B”. The sum of boxes of the FTLD-Clinical Dementia Rating (FTLD-CDR) as a validated score for measuring progression of functional decline [62] was available for all patients. The study was approved by the local ethics committees (Ethics Committee of the University of Ulm, reference 39/11 and Institutional Review Board of Azienda Sanitaria Locale Lecce, report n. 6, 25 July 2017, respectively), and written informed consent was obtained from each participant or the primary caregiver in accordance with the Declaration of Helsinki.

Among the participants of cohort A, the 30 nfPPA, 25 svPPA patients, and 15 controls underwent MR scanning on a 3.0T scanner (Ulm: Allegra, Siemens Medical). Among the participants of the cohort B, 3 nfPPA, 4 svPPA patients, and 24 controls underwent MR scanning on a 3.0T scanner (Tricase: Philips Ingenia). DTI and T1-weighted data were acquired using center-specific protocols. The DTI protocol used for cohort A consisted of 31 gradient directions (GD), including one *b* = 0 GD (80 slices, 112 × 128 pixels; slice thickness was 2.0 mm, in-plane pixel size was 2.0 × 2.0 mm^2^). The echo time (TE) and repetition time (TR) were 88 and 11,100 ms; *b* was 1000 s/mm^2^. The DTI protocol used for cohort B consisted of 65 GD, including one *b* = 0 GD (60 slices, 96 × 96 pixels; slice thickness was 2.5 mm, in-plane pixel size was 2.5 × 2.5 mm^2^); TE and TR were 85 and 6852 ms; *b* was 1000 s/mm^2^.

The structural Fast-Field Echo (FFE) T1-weighted data acquired for cohort A included 144 sagittal slices with 1.2 mm thickness, 1.0 × 1.0 mm^2^ in-plane resolution in a 256 × 248 matrix, TE = 4.2 ms, and TR = 1640 ms. The FFE T1-weighted sequences for cohort B were acquired with the following parameters: 200 slices, TR = 8.2 ms, TE = 3.8 ms, field of view = 256 × 256 mm^2^, flip angle = 8°, and voxel size = 1 mm^3^ isotropic.

Among the participants from the cohort A, 10 nfPPA and 6 svPPA patients had a follow-up scan including DTI with a time-interval of in average 12 months. The average time-interval between baseline and follow-up MRI was 11.80 ± 1.40 months for nfPPA patients and 12.20 ± 1.60 months for svPPA patients, with no significant difference between groups (*p* = non-significant). The remaining patients and controls were not available for a second MRI investigation due to progression of the clinical symptoms and associated inconveniences.

### Microstructural MRI analysis—DTI—standardized data pre-processing

The DTI analysis software *Tensor Imaging and Fiber Tracking* (TIFT) [63, 64] was used for post-processing and statistical analysis.

#### MNI normalization

After motion correction of individual DTI data sets, baseline and follow-up DTI data were aligned by fitting the (*b* = 0) volumes to minimize intensity differences, using halfway linear registration matrices to avoid baseline data bias [65]. Subsequently, baseline and follow-up data underwent stereotaxic Montreal Neurological Institute (MNI) transformation using identical parameters. After quality control including visual examination, 4 nfPPA and 2 svPPA subjects were discarded due to low data quality.

Spatial normalization to the MNI stereotaxic standard space was performed iteratively [66]. This process utilized a study-specific (*b* = 0) template and an additional FA template for the second iteration, as the FA template provides greater contrast than (*b* = 0) images [67]. The correlation between individual FA maps and the FA template exceeded 0.7 after two iterations, so the iterative process was halted [68]. Directional information during the normalization process was preserved using techniques described by Alexander et al. [69]. Eventually, 29 nfPPA and 27 svPPA patients were included in the analyses of the current study. An isotropic three-dimensional 8 mm full-width at half-maximum Gaussian smoothing filter was applied to the individual normalized FA maps. This filter size, approximately two-to-three times the recording voxel size depending on the protocol, offers a good balance between sensitivity and specificity [64].

#### Inter-center correction

Fractional anisotropy maps from the different protocols were corrected for age using regression models based on datasets from 15 and 24 controls, separately for the two contributing centers. Subsequently, FA maps of patients with nfPPA, svPPA, and controls were harmonized for center by applying respective 3-D correction matrices (linear first-order correction). These 3-D correction matrices were derived as linear adjustments based on differences in the DTI scans of controls from each center [52, 70]. No residual site effects could be detected after inter-center correction.

#### Definition of tract structures

To apply group-based fiber tracking (FT) algorithms [69], an averaged template DTI dataset was generated from 24 controls using the same DTI protocol. This involved arithmetic averaging of the MNI-transformed data. Eigenvectors and eigenvalues were calculated for each voxel position, representing the average of the 24 controls. Only controls were used to avoid bias from pathology-induced alterations. Directional information from each dataset was preserved during normalization and incorporated into template creation [69].

This averaged DTI dataset from controls was then utilized to identify pathways for defined TOIs for the four groups of patients. Given that no neuropathological staging hypothesis has been put forward for individual clinical PPA syndromes due to the heterogeneity of their underlying neuropathology [10, 71], the previous DTI studies in nfPPA [13, 17, 18, 29, 33, 42, 72, 73] and svPPA [13, 17, 18, 29, 33, 42, 72–74] were used as references for TOI definitions. White matter tracts were selected if they were described to be affected in >75% of the selected neuroimaging papers [75].

A seed-to-target approach was used [76, 77]. Seed regions were defined for both the seed and target regions. For fiber tracking, only voxels with an FA value above 0.2 were considered. A modified probabilistic streamline tracking approach, which accounts for the directional information of neighboring fiber tracts, was used for fiber tracking [69]. All fiber tracts originating in the seed regions and terminating in the target regions of the respective pathway defined the corresponding TOI.

The technique of tract-wise fractional anisotropy statistics (TFAS) [63] was employed to quantify the tractography results using the TOIs. The FA values of the specific tracts were arithmetically averaged for each stereotaxically normalized DTI dataset of each subject. The following TOIs were thus defined for the nfPPA and svPPA clinical syndromes: left and right uncinate fasciculus (UF), genu, section II, III IV and splenium of the corpus callosum (CC), left and right superior longitudinal fasciculus (SLF), left and right inferior longitudinal fasciculus (ILF), inferior fronto-occipitalis fasciculus (IFOF), cingulum, pontine projections, anterior thalamic radiation, corticostriatal projections, corticospinal tract (CST), optic radiation, fornix, and left and right tapetum.

### Structural MRI analysis: atlas-based volumetry

The T1-weighted data were processed using MATLAB (version R2014b, The Mathworks, USA) and the Statistical Parametric Mapping 12 (SPM12) software (Wellcome Trust Center for Neuroimaging, London, UK, www.fil.ion.ucl.ac.uk/spm), following a standardized processing pipeline for ABV; ABV has already been successfully employed in cross-sectional and longitudinal studies [54, 56–58, 78]. The processing steps included: (1) segmentation into gray matter, white matter, and cerebrospinal fluid (CSF) compartments, (2) stereotaxic normalization into MNI space, and (3) volumetric assessment using voxel-by-voxel multiplication and subsequent integration of normalized modulated component images (GM, WM, or CSF) with predefined masks from various brain atlases.

To improve the quality of atlas space mapping, high-dimensional registration methods were introduced, showing intrascanner variability of volumetric results to be less than 1% for most investigated structures [55]. All volumetric results were linearly standardized to the mean intracranial volume (ICV) of controls. Group-level differences were tested for significance after correction for multiple comparisons.

Two controls and 1 subject with nfPPA had to be excluded from the analysis as their T1-weighted data were compromised by artifacts.

A series of standard cortical and subcortical SOIs were chosen for ABV analysis [78]. These were the cerebrum gray and white matter, the frontal lobes, the temporal lobes, the parietal lobes, the occipital lobes, the insulae; the cerebellum, the brainstem, the left and right hippocampus, the left and right amygdala, the left and right caudate, the left and right putamen, and the left and right thalamus.

### DTI post-processing

#### Whole brain-based voxel-wise statistics at the group level

Whole brain-based spatial statistics (WBSS) [52, 79] was used to calculate cross-sectional differences in FA maps. Statistical comparisons between the patients with nfPPA (*n* = 29), svPPA (*n* = 27) and 39 controls were performed voxel-wise using the Welch’s test, with the FA threshold set at 0.2 [79, 80]. Statistical results were corrected for multiple comparisons using the false discovery rate (FDR) algorithm at *p* < 0.05 [81]. Additionally, Type 1 error was further reduced by applying a spatial correction algorithm that eliminated isolated voxels or small clusters of voxels within the size range of the smoothing kernel, resulting in a cluster-size threshold of 256 voxels (256 mm^3^).

WBSS was also performed to calculate longitudinal differences in FA maps. White matter FA values were corrected for age [82], and statistical voxel-wise comparisons of FA for 10 patients with nfPPA and 6 patients with svPPA were performed versus the 39 control datasets acquired at baseline. The results were corrected for multiple comparisons using the FDR algorithm at *p* < 0.05, with an additional cluster-size correction for type 1 error as previously described.

#### Cross-sectional tract-wise comparison at the group level

To quantify the directionality of the underlying tract structures, the technique of tract-wise FA statistics (TFAS) [63] was applied. Age and scanner-corrected FA maps from baseline scans of patients with nfPPA (*n* = 29), svPPA (*n* = 27), and 39 controls were used to calculate mean FA values for the investigated tracts. Cross-sectional comparisons of mean FA values between patients and controls were performed.

#### Cross-sectional region-wise comparison at the group level

For the detection of volumetric alterations, group-level differences in ABV of nfPPA and svPPA compared to controls, respectively, and nfPPA compared to svPPA, were assessed for statistical significance following FDR correction for multiple comparisons. Z-scores were calculated as the difference between the subject’s mean and the control group’s mean, divided by the control group’s standard deviation.

#### Longitudinal tract-wise comparison at the group level

Fractional anisotropy maps from patients with nfPPA (*n* = 10) and svPPA (*n* = 6) and controls (*n* = 39) who had received at least one follow-up scan were analyzed to calculate group-averaged differences in the staging-associated tracts.

#### Longitudinal region-wise comparison at the group level

For volumetric SOIs, group-level differences for longitudinal data were assessed for statistical significance versus controls, following correction for multiple comparisons with FDR. Z-scores were calculated as the difference between the subject’s mean and the control group’s mean, divided by the control group’s standard deviation.

#### Cross-sectional correlation of FA maps to clinical scores

Fractional anisotropy maps from 29 patients with nfPPA and 27 patients with svPPA were voxel-wise correlated to the sum of boxes of the FTLD-CDR scores. Results were corrected for multiple comparisons with the FDR and cluster-size approach described above. For the correlation analyses, spherical regions of interest (ROIs) were placed bi-hemispherically within the peak results clusters of WBSS. The average FA values underlying the respective fiber tracts (as estimated by TFAS analysis) were also correlated to sum of boxes of the FTLD-CDR scores.

### Classification by machine learning: random forest model

For the selection of the most important features to distinguish patients with nfPPA and svPPA from controls, two random forest algorithm were implemented. This model allows an understanding of the hierarchical order of the selected features by means of the Gini importance. As the model was trained using FA values from the TOIs employed in the TFAS analyses along with SOIs employed in the ABV analysis, its application was instrumental in identifying key SOIs and TOIs that significantly contributed to the classification task [83–85]. Furthermore, random forest models have been used successfully in a former study with a similar data structure combining DTI and T1-weighted data [86].

For model development, the dataset was divided into 80% training and 20% validation cohorts. Two controls and one subject with nfPPA had to be excluded from the analysis as their volumetric data were compromised by artifacts. The training cohort included 30 controls, 22 patients with nfPPA and 22 patients with svPPA. For validation the remaining 7 controls, 5 patients with nfPPA and 5 patients with svPPA were used. To reduce the risk of overfitting because of the limited sample size, a fivefold cross-validation was applied [87]. This means that each model for a defined feature selection was implemented five times: in each iteration, training was done on fourfold (80% of the data), and validation was performed out on the remaining fold (20%). This iteration was repeated until each fold had served once as the validation set. For all models in an iteration, cross-validated average accuracy, sensitivity, and specificity was calculated (Supplementary Table 7).

The random forest classifiers were implemented using the Scikit-learn library [88]. They were configured with the following key hyperparameters: the number of trees was fixed at 100, the Gini index was used as the splitting criterion, and the maximum depth of the trees was set to 4. Additionally, the minimum number of samples required to split a node was set to 2, and the minimum number of samples required at a leaf node was set to 1. For each split, a random subset of features (square root of the total number) was considered (max_features = ‘sqrt’), and bootstrap sampling was enabled. All models were trained using a fixed random state to ensure reproducibility. Feature selection was performed iteratively by removing the least important features based on the Gini index, retaining only those that maintained or improved classification accuracy [83, 87].

## Results

### Demographic data

The patients’ mean age (nfPPA, 60.4 ± 5.0; svPPA, 62.4 ± 10.4) was not significantly different from that of the controls (57.9 ± 11.6). Fifteen/29 (52%) and 15/27 (55%) of the subjects were male in the nfPPA and in the svPPA groups, respectively, while 7/39 (18%) of the subjects were male in the control group. The subjects were significantly different in mean MMSE scores (controls, 27.5 ± 1.21; nfPPA, 25 ± 3.6; svPPA, 22.7 ± 5.3; *p* < 0.001 for both comparison). Furthermore, the two subgroups of controls differed significantly in mean MMSE scores (SCD, 27.6 ± 1.2; healthy subjects, 29.6 ± 0.5; *p* < 0.001), although this difference was not clinically significant, as all scores were above the commonly accepted threshold of 24 for cognitive impairment. The mean sum of boxes of the FTLD-CDR scores was 3.4 ± 2.3 in the nfPPA group and 5.1 ± 4.2 in the svPPA group.

For DTI-based FA analyses as well as for GM volumetry analyses, no significant differences between SCD and healthy controls could be detected (Supplementary Fig. 1, Supplementary Tables 8 and 9).

### WBSS of cross-sectional microstructural differences

**Fig. 1:**
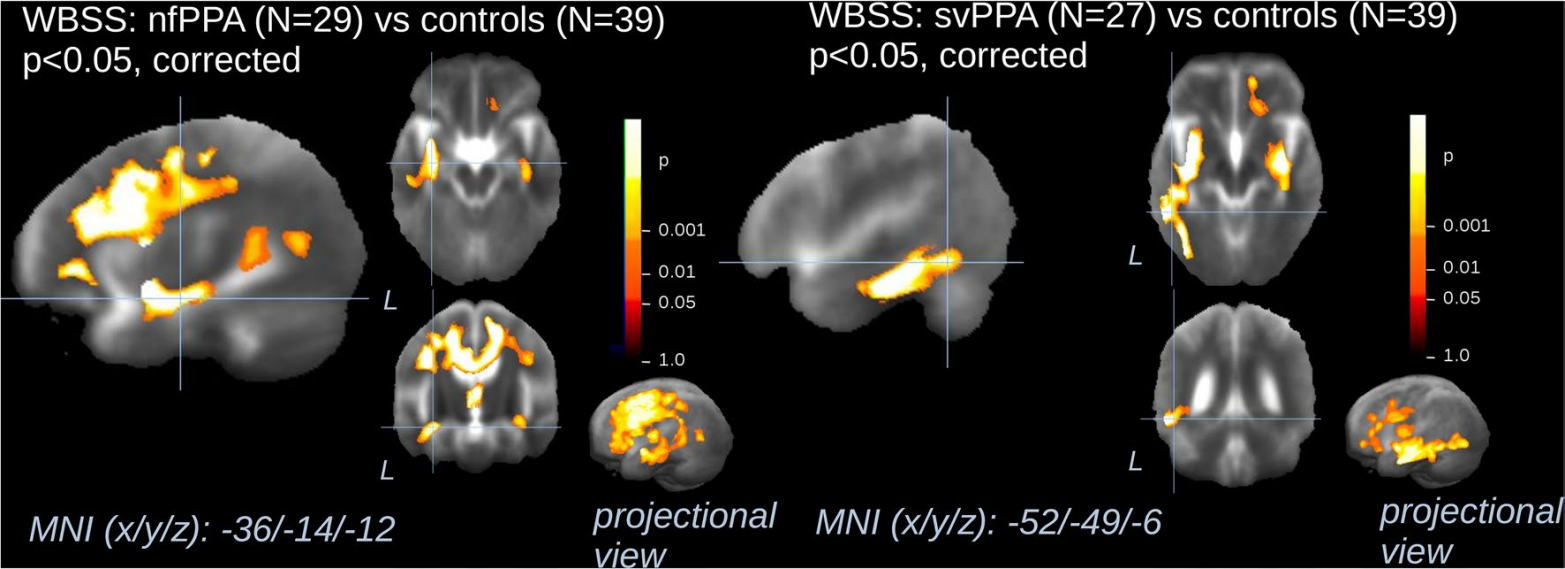
Whole brain-based spatial statistics for cross-sectional comparison of FA maps of patients with nfPPA and patients with svPPA vs controls. This figure shows slice-wise and projectional views of cross-sectional differences in baseline fractional anisotropy (FA) in 29 patients with non-fluent variant primary progressive aphasia (nfPPA, **A**) and 27 patients with semantic variant primary progressive aphasia (svPPA, **B**) vs 39 controls. Representative slices on the three axes demonstrate the main voxel clusters of results for the clusters for nfPPA (**A**) and svPPA (**B**). A projectional overlay reveals all clusters in a pseudo-3-D view (bottom right). Significance level is coded according to the color bar. Corrections for multiple comparisons were made with the false discovery rate at *p* < 0.05 and a cluster-size approach. Details of the corresponding clusters are shown in Tables 1 and 2. WBSS = whole brain spatial statistics; MNI = Montreal Neurological Institute

The group differences in white matter FA values between 29 patients with nfPPA and 39 controls demonstrated a significant decrease along the CC and in the white matter fibers of the frontal and of the temporal lobes (Fig. 1, Tables 1 and 2). Voxel-wise differences between the group of 27 patients with svPPA and controls predominantly demonstrated an FA decrease in the white matter bundles of the left temporal lobe (Fig. 1). Additional significant clusters were identified in the frontal lobes and in the anterior CC. In the putamen, bilaterally, an increase in FA values was observed. These findings were consistent with the alterations observed in nfPPA and svPPA-related TOIs (see Table 3).

**Table 1:** Whole brain-based spatial statistics for cross-sectional comparison of FA maps of patients with nfPPA (*n* = 29) vs controls (*n* = 39)

Anatomical location	L/R	*X*	*Y*	*Z*	No. of voxels
Frontal lobes/corpus callosum	L/R	±20	17	27	112,043
Left temporal lobe	L	−47	−48	7	12,082
Right temporal lobe	R	42	−23	−9	3481

This table shows details of the clusters where FA changes were detected in patients with nfPPA (*n* = 29) compared to controls (*n* = 39) by whole brain-based spatial statistics

*FA* fractional anisotropy**, ***nfPPA* non-fluent primary progressive aphasia, *L/R* left/right

**Table 2:** Whole brain-based spatial statistics for cross-sectional comparison of FA maps of patients with svPPA (*n* = 27) vs controls (*n* = 39)

Anatomical location	L/R	*X*	*Y*	*Z*	No. of voxels
Temporal lobe	L	−41	−1	27	19,251
Frontal lobe/corpus callosum	L/R	±7	5	25	15,430
Temporal lobe	R	38	−7	−6	6025
Basal ganglia	L	−27	−4	9	2626
Basal ganglia	R	23	0	10	1551

This table shows details of the clusters where FA changes were detected in patients with svPPA (*n* = 27) compared to controls (*n* = 39) by whole brain-based spatial statistics

*FA* fractional anisotropy**, ***svPPA* semantic variant of primary progressive aphasia, *L/R* left/right

**Table 3:** Cross-sectional fractional anisotropy (FA) differences in the white matter TOIs at group level

Tract of interest (TOI) analysis	nfPPA (*p* value)	svPPA (*p* value)
Left uncinate fasciculus	0.032	0.045
Right uncinate fasciculus	ns	ns
Genu of the corpus callosum	ns	ns
Splenium of the corpus callosum	ns	ns
Section II of the corpus callosum	<0.001	ns
Section III of the corpus callosum	ns	ns
Section IV of the corpus callosum	ns	ns
Left superior longitudinal fasciculus	0.029	ns
Right superior longitudinal fasciculus	ns	ns
Left inferior longitudinal fasciculus	0.036	<0.001
Right inferior longitudinal fasciculus	ns	ns
Inferior fronto-occipitalis fasciculus	0.005	0.006
Cingulum	<0.001	ns
Pontine projections	ns	ns
Anterior thalamic radiation	ns	ns
Corticostriatal projections	0.005	ns
Corticospinal tract	ns	ns
Optic radiation	ns	ns
Fornix	ns	ns
Left tapetum	ns	ns
Right tapetum	ns	ns

This table shows the differences in mean fractional anisotropy (FA) values at baseline between 29 patients with nfPPA, 27 patients with svPPA, and 39 controls. *p* values are reported if the difference between the mean volumetric and FA values of controls and patients with nfPPA and svPPA, respectively, was significant (*p* < 0.05, corrected for multiple comparisons)

*nfPPA* non-fluent variant of primary progressive aphasia, *svPPA* semantic variant of primary progressive aphasia, *TOIs* Tracts of Interest, *ns* non-significant

### Cross-sectional microstructural differences in the white matter TOIs at group level

Significant group differences in mean FA between patients with nfPPA and controls were observed in the TOIs (Table 3). These involved decreased FA values in the segment II of the CC (*p* < 0.001), left superior (*p* = 0.03) and inferior (*p* = 0.03) longitudinal fasciculus, left UF (*p* = 0.03), IFOF (*p* = 0.005), cingulum bundle (*p* < 0.001), and increased FA values in the corticostriatal projections (*p* = 0.005). Significant group differences in mean FA between 27 patients with svPPA and controls were observed for left temporal lobe WM bundles such as the ILF (*p* < 0.001) and the UF (*p* = 0.05), as well as the IFOF (*p* = 0.006).

### Cross-sectional structural differences in the gray matter SOIs at group level

In the ABV analysis, both nfPPA (*n* = 28) and svPPA (*n* = 27) patients exhibited significant volumetric reductions in comparison to controls (*n* = 37) in most of the investigated SOIs (z scores and *p* values are displayed in Table 4).

**Table 4:**
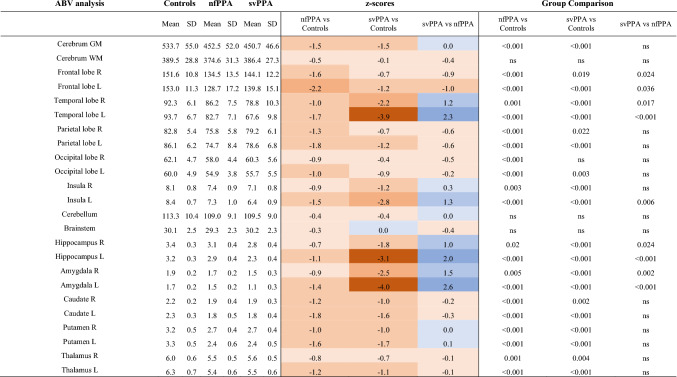
Cross-sectional volumetric differences in the SOIs at group level

This table shows the differences in SOI volumes (ml) at baseline between 29 patients with nfPPA, 27 patients with svPPA, and 39 controls. Mean and standard deviation are reported for each group. Z-scores were calculated as the difference between the subject’s mean and the control group’s mean, divided by the control group’s standard deviation. A 2-color scale was used to rank the z-scores. FDR-corrected *p* values are reported if significant

*SOIs* Structures of Interest, *ABV* atlas-based-volumetry, *nfPPA* non-fluent variant of primary progressive aphasia, *svPPA* semantic variant of primary progressive aphasia, *SD* standard deviation, *GM* gray matter, *WM* white matter, *ns* non-significant

In a direct comparison, nfPPA exhibited significantly higher volumetric reduction than svPPA at the level of the bilateral frontal lobe. Vice versa, svPPA showed significantly higher volumetric reduction than nfPPA in temporal lobes, the left insula, hippocampus, and amygdala (differences in z scores between the two syndromes as well as the *p* values for the direct comparison are displayed in Table 4).

### Longitudinal differences for WBSS and TOI-based FA values

**Fig. 2:**
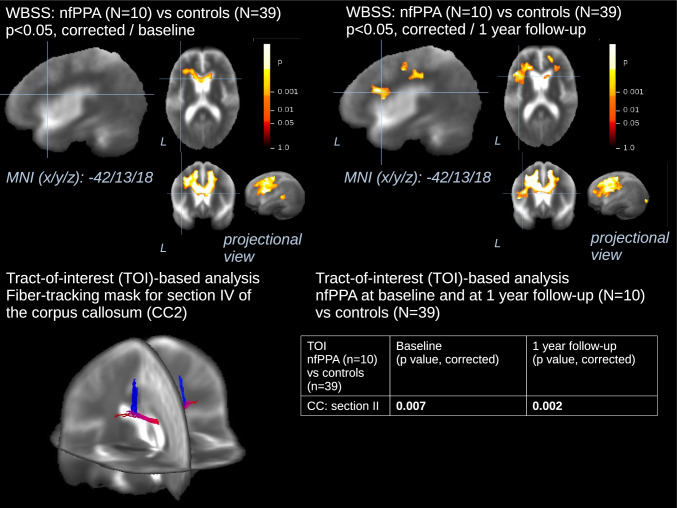
Whole brain-based spatial statistics (WBSS) and Tract-of-Interest (TOI)-based statistics for longitudinal comparison of FA maps of patients with nfPPA vs controls. This figure shows slice-wise and projectional views of WBSS (**A, B**) and TOI-based (**C, D**) longitudinal differences in FA in 10 nfPPA from baseline to 1-year follow-up vs 39 controls. The slices on the three axes are centered in the main cluster identified to show additional white matter involvement over the 1-year long follow-up, for the purpose of representation. A projectional overlay reveals all clusters in a pseudo-3-D view (**A, B**, bottom right). Significance level is coded according to the color bar. Corrections for multiple comparisons were made with the false discovery rate at *p* < 0.05 and a cluster-size approach. Details of the corresponding clusters at baseline and at 1-year follow-up are shown in Supplementary Tables 1 and 2, respectively. A 3-D representation of the tract where the difference in mean FA values had the higher degree of significance vs controls is shown (**C**). *P* values for the corresponding TOI-based statistics are displayed (D). WBSS = whole brain spatial statistics; TOI = Tract-of-Interest; MNI = Montreal Neurological Institute

**Fig. 3:**
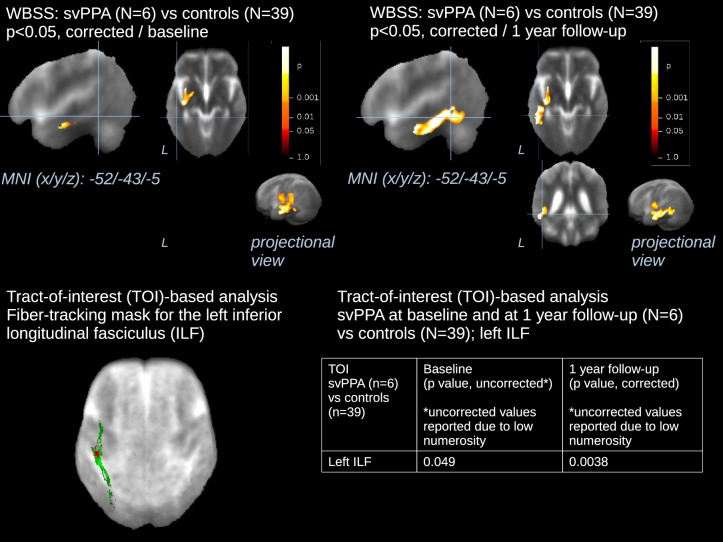
Whole brain-based spatial statistics (WBSS) and Tract-of-Interest (TOI)-based statistics for longitudinal comparison of FA maps of patients with svPPA vs controls. This figure shows slice-wise and projectional views of WBSS (**A, B**) and TOI-based (**C, D**) longitudinal differences in FA in 6 svPPA patients from baseline to 1-year follow-up vs 39 controls. The slices on the three axes are centered in the main cluster identified to show additional white matter involvement over the 1-year long follow-up, for the purpose of representation. A projectional overlay reveals all clusters in a pseudo-3-D view (**A, B**, bottom right). Significance level is coded according to the color bar. Corrections for multiple comparisons were made with the false discovery rate at *p* < 0.05 and a cluster-size approach. Details of the corresponding clusters at baseline and at 1-year follow-up are shown in supplementary Tables 3 and 4, respectively. A 3-D representation of the tract where the difference in mean FA values had the higher degree of significance vs controls is shown (**C**). *P* values for the corresponding TOI-based statistics are displayed (**D**). WBSS = whole brain spatial statistics; TOI = Tract-of-Interest; MNI = Montreal Neurological Institute

For the longitudinal WBSS analysis of voxel-wise FA values, an increase in the size of all the clusters could be observed in 10 patients with nfPPA vs 39 controls (Fig. 2, Supplementary Tables 1 and 2): longitudinal white matter degeneration could be observed along the corpus callosum and frontal lobes, with a prominent additional involvement of the left frontal lobe white matter. In the six patients with svPPA, longitudinal progression of microstructural damage mainly involved the left temporal lobe white matter (Fig. 3, Supplementary Tables 3 and 4). In detail, an antero-posterior progression of white matter changes along the left ILF could be observed. Additionally, an increase in FA values was observed for the bilateral putamen. These significant longitudinal alterations were *grosso modo* consistent with the findings observed in the nfPPA- and svPPA-related TOIs (Figs. 2 and 3). In detail, FA of the section II of the corpus callosum was significantly different between nfPPA patients and controls both at baseline (*p* = 0.007) and follow-up (*p* = 0.002), whereas the comparison of TOI-based FA values between six svPPA patients and 39 controls did not provide any result surviving statistical corrections, probably due to the low number of the sample.

### Longitudinal differences in SOI-based ABV analysis

In the ABV analysis, in the 10 nfPPA patients with follow-up data, a significant volumetric reduction from baseline could be appreciated for frontal lobes, the left temporal lobe, parietal lobes, the left insula, the right hippocampus, the left amygdala, and the bilateral putamen and thalamus. The highest variation in the volumetric z score were registered in the frontal lobes, the left temporal lobe, the bilateral putamen, and the left thalamus. In the svPPA cohort, significant volumetric reductions from baseline were localized in the left temporal lobe, the left insula, the left hippocampus, the left amygdala, and the left putamen. These were also the SOIs where the highest degree of variation in the z score was registered (z scores and *p* values are displayed in Supplementary Tables 5 and 6).

### Correlation of voxel-wise FA values with FTLD-CDR sum of boxes

**Fig. 4:**
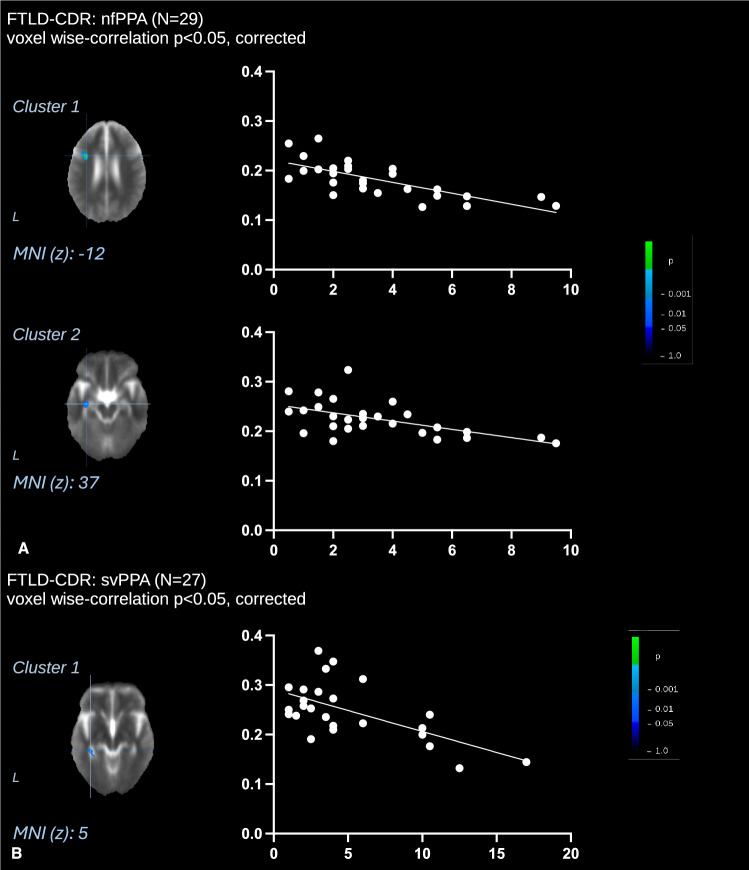
Whole brain-based spatial statistics (WBSS) for correlation of voxel-wise FA values with clinical scores. This figure displays slice-wise views of cross-sectional correlations of fractional anisotropy (FA, *y* axis) in 29 patients with nfPPA (**A**) and 27 patients with svPPA (**B**), respectively, with sum of boxes of the frontotemporal lobar degeneration modified-clinical dementia rating (FTLD-CDR, x axis) score. Main clusters are shown with Montreal Neurological Institute [MNI] coordinates of the peak voxel. The significance level is indicated by the color bar: cool colors represent a negative correlation. Corrections for multiple comparisons were applied using the false discovery rate at *p* < 0.05 and a cluster-size approach. Details for each cluster (correlation coefficient *R* and corrected *p* value, as well as size, expressed as no. of voxels) are displayed in Tables 5 and 6 for nfPPA and svPPA, respectively. WBSS = whole brain spatial statistics; MNI = Montreal Neurological Institute

A voxel-wise correlation (Fig. 4A**, **Tables 5 and 6) of FA maps from 29 patients with nfPPA with their scores of disease progression (sum of boxes of the frontotemporal lobar degeneration modified-clinical dementia rating, FTLD-CDR) showed significant negative correlations in the white matter of the left frontal lobe *c* < 0.001) and in the left temporal lobe (*p* = 0.002). A voxel-wise correlation of FA maps from 27 patients with svPPA with the sum of boxes of the FTLD-CDR score (Fig. 4B) revealed significant negative correlations in the white matter of the posterior left temporal lobe (*p* = 0.001).

**Table 5:** Cross-sectional correlations of FA maps of patients with nfPPA (*n* = 29) with clinical scores

Anatomical location	L/R	*X*	*Y*	*Z*	No. of voxels	Correlation (*R*; corrected *p* value)
Frontal lobe/corpus callosum	L	−35	6	37	763	−0.72; <0.001
Temporal lobe	L	−35	−16	−12	374	−0.56; 0.002

This table shows details of the clusters where FA changes were significantly correlated with sum of boxes of the FTLD-CDR score in patients with nfPPA (*n* = 29) by whole brain-based spatial statistics (Fig. 4A)

*FA* fractional anisotropy**, ***nfPPA* non-fluent primary progressive aphasia, *L/R* left/right

**Table 6:** Cross-sectional correlations of FA maps of patients with svPPA (*n* = 27) with clinical scores

Anatomical location	L/R	*X*	*Y*	*Z*	No. of voxels	Correlation (*R*; corrected *p* value)
Temporal lobe (posterior)	L	−38	−38	−5	1792	−0.61; 0.001

This table shows details of the clusters where FA changes were significantly correlated with sum of boxes of the FTLD-CDR score in patients with svPPA (*n* = 27) by whole brain-based spatial statistics (Fig. 4B)

*FA* fractional anisotropy**, ***svPPA* semantic variant of primary progressive aphasia, *L/R* left/right

### Random forest classification

**Fig. 5:**
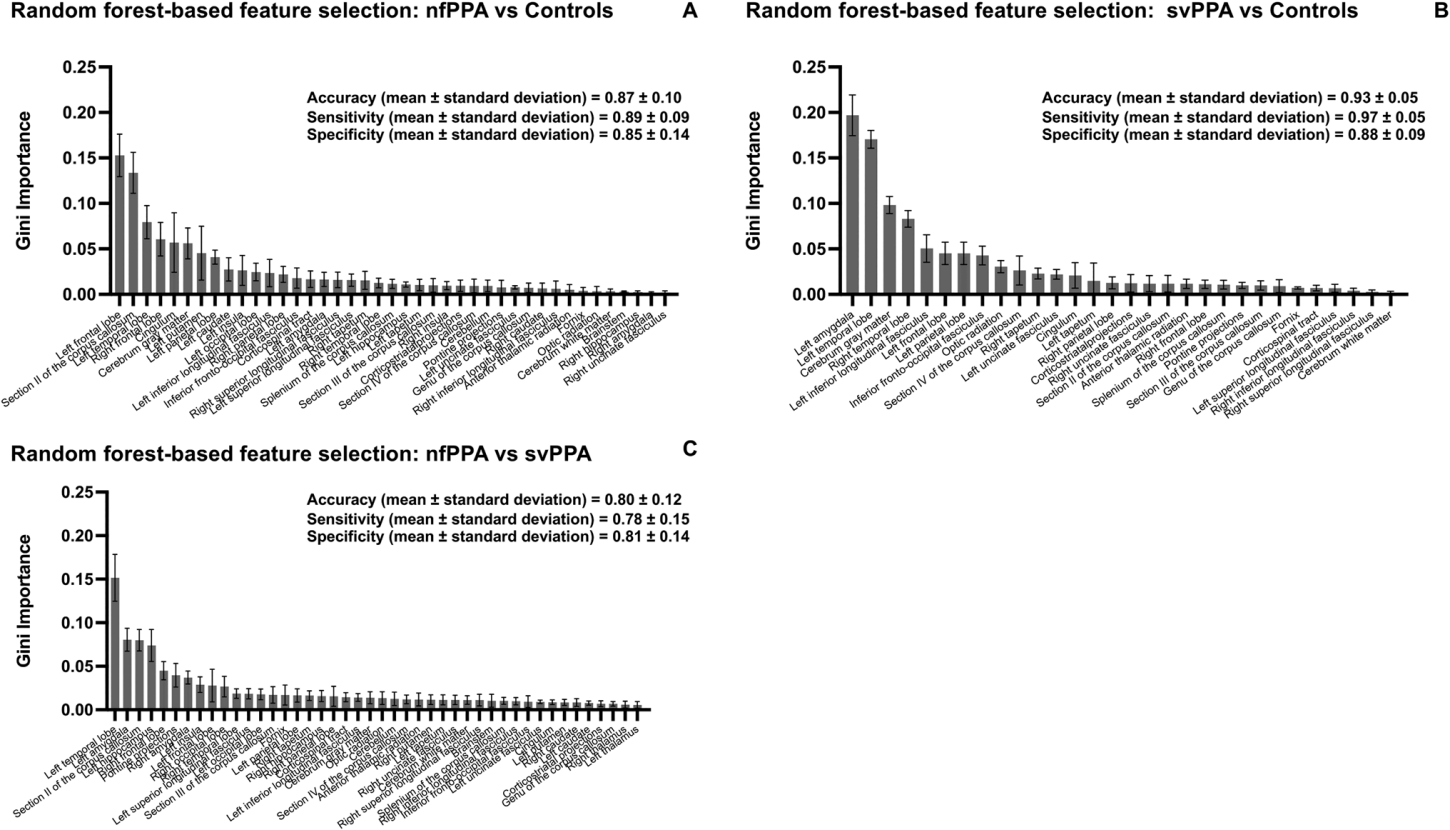
Classification of patients into diagnostic groups with a random forest algorithm. This illustration shows the Gini importance values derived from the random forest approach for classifying nfPPA (**A**) and svPPA (**B**) patients versus controls as well as patients with nfPPA vs patients with svPPA (**C**), using TOIs from TFAS and SOIs from ABV, as well as the averaged values for accuracy, sensitivity, and specificity between the fivefold calculations for nfPPA and svPPA compared to controls, respectively

To classify nfPPA and svPPA patients versus controls, a random forest method was used, including all the TOIs and SOIs employed in the study. The model provided an averaged accuracy of 0.87 ± 0.10 with a sensitivity of 0.89 ± 0.09 and a specificity of 0.85 ± 0.14 for the nfPPA group (Fig. 5A) and an accuracy of 0.93 ± 0.05 with a sensitivity of 0.97 ± 0.05 and a specificity of 0.88 ± 0.09 for the svPPA group (Fig. 5B). Furthermore, a classification between nfvPPA and svPPA provided a cross-validated accuracy of 0.80 ± 0.12 with a mean sensitivity 0.78 ± 0.15 and specificity of 0.81 ± 0.14 (Fig. 5C). The feature selection lead to a set of 44 features. The features with the higher Gini importance for the model served as a proof-of-concept validation for the a priori assumptions in selecting TOIs and SOIs. For the nfPPA group, the key regions were the left frontal lobe, section II of the corpus callosum, left temporal lobe, right frontal lobe, and the cingulum. For the svPPA group, the key regions included the left amygdala, left temporal lobe, cerebrum gray matter, right temporal lobe, the left ILF.

For the direct comparison between nfPPA and svPPA, the most relevant among these were: the left temporal lobe, amygdala, corpus callosum area II, and left hippocampus. The gini importance of the left temporal lobe was in every cross-validated fold the most important feature, while the other features differed in between every fold in their hierarchical order (Supplementary Table 7).

## Discussion

This study examined the role of multi-parametric structural imaging by DTI and ABV in monitoring the spatiotemporal dynamics of gray and white matter degeneration in the PPA variants nfPPA and svPPA. The cross-sectional analysis demonstrated distinct patterns of white matter degeneration for nfPPA and svPPA, further supported by widespread volumetric reductions in both groups as shown by ABV analysis. The longitudinal analysis documented a general substantial increase of previously observed clusters of frontal, callosal, and temporal white matter degeneration, with the progression of white matter damage occurring along tracts that were already affected at baseline in both the groups of patients. Voxel-wise correlations of sum of boxes of the FTLD-CDR scores with white matter FA changes yielded significant clusters, overlapping with sections of the tracts observed to be longitudinally involved in the PPA variants.

### Cross-sectional structural changes in nfPPA and svPPA

Compared to previous studies using unbiased voxel-wise DTI analyses to assess white matter alterations in PPA variants, our study included one of the largest PPA cohorts to date. Earlier investigations, such as Agosta et al. (*n* = 9 nfPPA, 7 svPPA) [72], Lam et al. (*n* = 10 nfPPA, 11 svPPA) [33] consistently identified the hallmark topographical dissociation of dorsal pathway involvement (i.e., SLF and callosal fibers) in nfPPA and left-lateralized ventral stream degeneration (notably the ILF and UF) in svPPA. Our findings are in line with this dissociation and further refine it. The results of this study for nfPPA substantially agree with the previous data-driven conceptualizations of nfPPA as a primarily SLF/callosal disease [12, 17, 33, 38, 41, 72, 89]. However, our results also support observations of mild anterior-temporal fiber damage [17, 41]. By adding the specificity of tract-based analyses to the spatial resolution of voxel-wise approaches, we were able to identify additional involvement in the CST and cingulum. While the involvement of the former has been previously documented [90] in nfPPA, the latter has been only recently implicated in nfPPA-associated patterns by a study employing radiomics [91]. In svPPA, we confirmed a previously observed [33, 72] strongly left-lateralized pattern centered on the anterior-temporal lobe, with robust involvement of the ILF and UF. Compared to previous studies [42], our tract-oriented approach combined with voxel-wise precision allowed better spatial localization and enhanced statistical sensitivity for the reported findings compared to some of the previous studies, contributing to a more distinctive characterization of a variant-specific white matter signature. In more detail, our findings do not support interpretations [17, 23] of widespread white matter involvement in early stage svPPA. Instead, they confirm that the semantic hubs in the anterior-temporal lobes are the primary focus of degeneration.

Corticostriatal involvement in PPA has only rarely [90] been reported in studies employing DTI to investigate disease-specific patterns in these syndromes. Our data indicate an FA increase in basal-ganglia projections in both nfPPA (TOI approach only) and svPPA (voxel-wise approach only), respectively. Previous studies on bvFTD and amyotrophic lateral sclerosis have also shown that FA increases in regions with a high density of crossing fibers, likely reflecting a technique-inherent pseudo-increase rather than a genuine preservation of white matter integrity [37, 76]. Although these findings lack coherence between TOI and voxel-wise approaches, they align with the previous studies on atrophy patterns in PPA variants [92], bvFTD [37, 57], and other tauopathies with frontal involvement such as corticobasal degeneration [93, 94] and progressive supranuclear palsy [95]. Notably, in svPPA-tau cases, more striatal atrophy has been reported compared to svPPA-TDP43 [96], while extrapyramidal motor signs were more frequently observed in nfPPA-tau than in nfPPA-TDP43 [96]. Collectively, these findings, together with the results of this study, highlight the early clinical and anatomical involvement of fronto-striatal structures in both svPPA and nfPPA. Whether this pattern is a manifestation of an underlying tauopathy needs to be confirmed by specific correlations between in vivo neuroimaging and *post-mortem* pathological data.

Atlas-based volumetric analyses confirmed the observed microstructural alterations, revealing that patients with nfPPA exhibited significantly more atrophy in the frontal lobes compared to those with svPPA, whereas svPPA patients showed more pronounced atrophy in the temporal lobes. These findings are consistent with the previous volumetric gray matter studies on PPA variants which have identified predominant frontal involvement in nfPPA [92] and left temporal atrophy in svPPA [57, 92, 97, 98]. Furthermore, svPPA patients demonstrated higher atrophy than nfPPA in the left insula, as well as in the bilateral hippocampus and amygdala. The more severe degeneration of the latter aligns with the more pronounced microstructural loss in svPPA documented by TFAS and WBSS at the level of the ILF and UF, which have been shown to connect the hippocampus with the temporo-parietal lobes [99] and the amygdala with the frontal lobes [100, 101], respectively. Taken together, these findings highlight the centrality of limbic disconnection in the pathogenesis of svPPA [97, 102]. While definitive in vivo differentiation of underlying pathology remains elusive, our findings may have speculative implications for clinico-pathological correlations. The prominent involvement of limbic structures and their associated tracts in svPPA may reflect a selective vulnerability of these regions to TDP-43 type C proteinopathy, the pathology most commonly associated with this variant. This is consistent with imaging evidence showing greater hypothalamic volume loss in TDP-43-related syndromes compared to tauopathies[103]. Although this interpretation remains speculative, it highlights the potential of structural MRI not only for phenotypic classification but also for probing disease mechanisms. Future studies incorporating longitudinal and pathology-confirmed cohorts will be critical to further clarify these associations.

Finally, a random forest model was tested to accurately classify patients into their respective diagnostic groups according to their TFAS-based FA and ABV-based volumetric values in TOIs and SOIs, respectively (Fig. 5). In more detail, we could demonstrate that, despite being trained with a relatively small number of individual datasets, a random forest model achieved a moderately good degree of accuracy when it was instructed to classify patients compared to controls as well as to discriminate between the two groups of patients with nfPPA and svPPA.

The results of the random forest analysis demonstrate strong alignment between the TOIs and SOIs that proved to reach the highest statistical significance in the group comparisons between patients and controls. The features with the highest Gini importance that identified nfPPA and svPPA patients compared to controls were different, further highlighting distinct patterns of feature relevance for classification. On the one hand, the five most important features for classification of subjects into the nfPPA group were all localized in the CC and frontal lobe areas (except for the left temporal lobe), with a strikingly high Gini importance for the left frontal lobe and the anterior CC (section II). On the other hand, the classification of subjects into the svPPA group heavily relied on the left amygdala and the left temporal lobe/ILF.

The prominence of these findings aligns with our TFAS and ABV results, supporting the validity of our a priori selection of the tracts. Furthermore, the fact that both TOIs (derived from TFAS) and SOIs (derived from ABV) were included among the most important features for classification reinforces the notion that a multi-parametric approach is needed in future structural neuroimaging studies on FTLD (Fig. 5).

### Longitudinal structural changes in nfPPA and svPPA

A few longitudinal DTI studies on PPA variants included a voxel-wise assessment in their protocol [33, 34, 39]. These documented a longitudinal reduction of FA in white matter fiber bundles in PPA variants, as observed in our study. The increase of the differences from controls in both extent of the clusters (Supplementary Tables 1–4) and significance for the involved TOIs (Figs. 2 and 3) supports the hypothesis of trans-axonal spreading of FTLD pathology [44]. What our longitudinal voxel-wise results strikingly capture, confirmed by TFAS, is the prominence of the antero-posterior progression along the left ILF in svPPA, overshadowing changes in other tracts. This finding refines previous DTI investigations in 19 and 11 svPPA patients, respectively, that found a less left-lateralized and localized pattern[33, 39]. Furthermore, it aligns with ABV [57, 92] studies and supports clinico-anatomical correlations between the worsening of semantic deficits and left, but not right, temporal lobe atrophy [104]. Our results, differently than previous studies that included both nfPPA and svPPA patients in their longitudinal voxel-wise assessment [33, 39] managed to highlight a divergent propagation of white matter changes in svPPA and nfPPA. The most prominent difference between our and previous studies [33, 34, 39] concerns the laterality of FA longitudinal changes in the temporal lobes, with the markedly left-lateralized pattern of our findings apparently at odds with the bilateral pattern of changes previously reported. This bilateral involvement may be attributed to differences in disease severity. As the median age in our longitudinal svPPA cohort is 4–5 years lower than in the previous studies [33, 34, 39], the right temporal lobe could be still spared in our sample of patients. This finding supports the hypothesis that the progression from the left to the right hemisphere is dependent on age and disease stage.

The absence of such a localized effect in our longitudinal nfPPA data is almost invariably corroborated by the previous DTI [33, 39, 105] and ABV [92] studies that report a widespread frontal and callosal involvement, as well as by our volumetric longitudinal data which document progression of atrophy in insula, temporo-parietal lobes, and subcortical structures (thalamus and amygdala). This more diffuse pattern can be attributed to the higher pathological heterogeneity of nfPPA. Specifically, DTI studies have identified distinct patterns associated with different proteinopathies: nfPPA-tau is associated with significant changes in the superior SLF and CC [96], whereas nfPPA-TDP43 was detected to present left frontal regions alterations [106]. Given these findings, our DTI-FA data likely represent a mix of these patterns, reflecting the overlap of tau- and TDP43-driven pathologies in nfPPA. However, the relative uniformity in longitudinal findings in svPPA [33, 34, 39] could be justified by a relative TDP-43 pathological homogeneity underlying this syndrome [107]. The consistent changes observed in the anterior corpus callosum in both our study and previous voxel-wise analyses in nfPPA [17, 33, 41, 72] and, to a minor degree, in svPPA [17, 41] further suggest that this progression occurs via callosal crossing fibers [17, 39, 41]. Finally, longitudinal basal-ganglia involvement was confirmed by our data in svPPA but not in nfPPA. Conversely, ABV revealed progression of striatal degeneration in both syndromes. Future studies specifically targeting corticostriatal white matter projections should disentangle whether there is a differential progression involving corticostriatal connectivity in the two syndromes.

### Correlation analyses

Significant correlations between microstructural alterations and clinical progression scores (sum of boxes of the FTLD-CDR) were identified in both nfPPA and svPPA groups. Specifically, the voxel-wise correlation analysis demonstrated that lower FA values in the white matter of the left frontal lobe were significantly associated with functional decline in nfPPA patients; additionally, smaller but significant clusters were observed in the left temporal lobe for both patients with nfPPA and svPPA. These findings are consistent with the critical role of frontal lobe white matter in executive and language functions commonly impaired in both nfPPA and svPPA and with the established role of temporal regions in semantic processing [33, 38, 39]. These findings further support the hypothesis of a topographically distinct propagation pattern of degeneration along key white matter pathways, consistent with the clinico-anatomical correlations observed in these syndromes.

### Limitations

The results of this study have to be considered in the context of several limitations. First, the sample size for longitudinal analyses was relatively small (although in a reasonable range for imaging studies to obtain statistically significant results and adequate, given the rarity of these diseases [108]). However, standardized DTI acquisition protocols have been shown to yield reliable and reproducible results even in studies with limited sample sizes, supporting the robustness of our findings despite the reduced cohort [109]. This allows us to generalize the significance of our findings, despite acknowledging that future studies should replicate our investigations with a bigger pool of subjects. Second, we acknowledge that the limited availability of follow-up MRI data may have introduced a drop-out bias, potentially favoring the inclusion of patients with milder disease progression and better clinical stability. Third, our phenotyping approach was limited to the first-level classification of PPA into the traditionally recognized semantic and non-fluent variants. Nonetheless, recent advancements emphasize the importance of a more detailed categorization of progressive language deficits, incorporating newly defined sub-syndromes to better capture the heterogeneity of these conditions. [45]. On the one hand, nfPPA has been recently distinguished in (at least) two subtypes whose core features are agrammatism [110] and speech apraxia [111, 112]. However, although TDP-43 has been sporadically detected in autoptic studies of primary progressive apraxia of speech patients [113, 114], a tauopathy remains the most frequently detected pathology underlying all non-fluent PPA variants [113, 115]. On the other hand, symptomatologic overlap between svPPA and a right temporal variant of FTD has been documented [116]. Some patients who were retrospectively included in this study could have been currently categorized as being affected by a right temporal variant of FTD (rtvFTD) whose presentation also includes subtle language deficits [116], but the underlying pathology of rtvFTD is believed to predominantly involve TDP-43, with a reported frequency as high as 90%, similar to that observed in svPPA [117, 118]. Given the pathological uniformity within the nfPPA and temporal lobe PPA syndromes, we are confident that, while deeper clinical phenotyping would undoubtedly enhance the precision of neuroimaging investigations, it would not have significantly altered the outcomes of our study. Furthermore, on a qualitative assessment of our MRI scans, only one subject could be found to show a higher degree of atrophy in the right rather than left temporal lobe in the svPPA group. Although our study did not include patients with logopenic PPA, this does not constitute a limitation, as the diagnostic workup for logopenic PPA relies mainly on CSF or blood-based biomarkers to demonstrate Alzheimer’s disease pathology [119], whereas neuroimaging remains the primary in vivo tool for the diagnosis of FTLD-spectrum variants such as nfPPA and svPPA.

Finally, we focused exclusively on FA as the primary metric of microstructural white matter damage. This decision is supported by prior studies from our group which have demonstrated the replicability, reliability, and stability of FA measurements in multisite settings using standardized acquisition protocols [52, 53].

## Conclusions

In conclusion, our study demonstrates the complementary roles of DTI and ABV in characterizing the structural and microstructural changes underlying nfPPA and svPPA. These techniques identified distinct patterns of degeneration and support attempts of early differential diagnosis, an approach that may be further refined through the broader application of machine learning models, as preliminarily demonstrated in this study. Furthermore, our study confirms the suitability of DTI and ABV for longitudinal tracking of disease progression in vivo. Our findings suggest that, in both PPA variants, neurodegeneration propagates along axonal pathways between functionally connected regions rather than spreading by chance or by contiguity between adjacent but functionally unrelated brain areas. Finally, our findings contribute to the growing body of evidence supporting variant-specific patterns of structural neuroimaging alterations in PPA, reinforcing the clinical utility of early, biomarker-informed diagnosis, an approach that may be further enhanced by large-scale implementation of machine learning models, as preliminarily explored in this study.

Future studies with larger longitudinal cohorts are essential to further validate these observations as well as to confirm potential clinic-pathological speculations (such as a possible TDP43-preferential involvement of limbic structures), refining our understanding of the distinct mechanisms driving progression in nfPPA and svPPA.

## Supplementary Information

Below is the link to the electronic supplementary material.

Supplementary file1 (DOCX 147 KB)

## Data Availability

The dataset used (fully anonymized) and analyzed during the current study will be made available by the corresponding author upon reasonable request by qualified researchers.

## References

[1] Grossman M, Seeley WW, Boxer AL et al (2023) Frontotemporal lobar degeneration. Nat Rev Dis Primers 9:1–19. 10.1038/s41572-023-00447-0 37563165 10.1038/s41572-023-00447-0

[2] MacKenzie IRA, Neumann M, Bigio EH et al (2010) Nomenclature and nosology for neuropathologic subtypes of frontotemporal lobar degeneration: An update. Acta Neuropathol 119:1–4. 10.1007/S00401-009-0612-2/TABLES/1 19924424 10.1007/s00401-009-0612-2 PMC2799633

[3] Irwin DJ, Cairns NJ, Grossman M et al (2015) Frontotemporal lobar degeneration: defining phenotypic diversity through personalized medicine. Acta Neuropathol 129:469–491. 10.1007/S00401-014-1380-1/METRICS 25549971 10.1007/s00401-014-1380-1 PMC4369168

[4] Rascovsky K, Hodges JR, Knopman D et al (2011) Sensitivity of revised diagnostic criteria for the behavioral variant of frontotemporal dementia. Brain 134:2456–2477. 10.1093/BRAIN/AWR179 21810890 10.1093/brain/awr179 PMC3170532

[5] Gorno-Tempini ML, Hillis AE, Weintraub S et al (2011) Classification of primary progressive aphasia and its variants. Neurology 76:1006–1014. 10.1212/WNL.0B013E31821103E6/SUPPL_FILE/GORNO.PDF 21325651 10.1212/WNL.0b013e31821103e6 PMC3059138

[6] Mesulam MM, Wieneke C, Thompson C et al (2012) Quantitative classification of primary progressive aphasia at early and mild impairment stages. Brain 135:1537–1553. 10.1093/BRAIN/AWS080 22525158 10.1093/brain/aws080 PMC3577099

[7] Wicklund MR, Duffy JR, Strand EA et al (2014) Quantitative application of the primary progressive aphasia consensus criteria. Neurology 82:1119–1126. 10.1212/WNL.0000000000000261 24598709 10.1212/WNL.0000000000000261 PMC3966800

[8] Giannini LAA, Irwin DJ, Mcmillan CT et al (2017) Clinical marker for Alzheimer disease pathology in logopenic primary progressive aphasia. Neurology 88:2276–2284. 10.1212/WNL.0000000000004034/SUPPL_FILE/SUPPLEMENTAL_DATA.DOCX 28515265 10.1212/WNL.0000000000004034 PMC5567322

[9] Giannini LAA, Xie SX, McMillan CT et al (2019) Divergent patterns of TDP-43 and tau pathologies in primary progressive aphasia. Ann Neurol 85:630–643. 10.1002/ANA.25465 30851133 10.1002/ana.25465 PMC6538935

[10] Mesulam MM, Weintraub S, Rogalski EJ et al (2014) Asymmetry and heterogeneity of Alzheimer’s and frontotemporal pathology in primary progressive aphasia. Brain 137:1176–1192. 10.1093/BRAIN/AWU024 24574501 10.1093/brain/awu024 PMC3959558

[11] Mcmillan CT, Avants BB, Cook P et al (2014) The power of neuroimaging biomarkers for screening frontotemporal dementia. Hum Brain Mapp 35:4827–4840. 10.1002/HBM.22515 24687814 10.1002/hbm.22515 PMC4107021

[12] Galantucci S, Tartaglia MC, Wilson SM et al (2011) White matter damage in primary progressive aphasias: a diffusion tensor tractography study. Brain 134:3011–3029. 10.1093/BRAIN/AWR099 21666264 10.1093/brain/awr099 PMC3187537

[13] Zhang Y, Tartaglia MC, Schuff N et al (2013) MRI signatures of brain macrostructural atrophy and microstructural degradation in frontotemporal lobar degeneration subtypes. J Alzheimers Dis 33:431–444. 10.3233/JAD-2012-121156 22976075 10.3233/JAD-2012-121156 PMC3738303

[14] Daianu M, Mendez MF, Baboyan VG et al (2016) An advanced white matter tract analysis in frontotemporal dementia and early-onset Alzheimer’s disease. Brain Imaging Behav 10:1038–1053. 10.1007/S11682-015-9458-5/TABLES/3 26515192 10.1007/s11682-015-9458-5 PMC5167220

[15] Borroni B, Brambati SM, Agosti C et al (2007) Evidence of white matter changes on diffusion tensor imaging in frontotemporal dementia. Arch Neurol 64:246–251. 10.1001/ARCHNEUR.64.2.246 17296841 10.1001/archneur.64.2.246

[16] Matsuo K, Mizuno T, Yamada K et al (2008) Cerebral white matter damage in frontotemporal dementia assessed by diffusion tensor tractography. Neuroradiology 50:605–611. 10.1007/S00234-008-0379-5/METRICS 18379765 10.1007/s00234-008-0379-5

[17] Schwindt GC, Graham NL, Rochon E et al (2013) Whole brain white matter disruption in semantic and nonfluent variants of primary progressive aphasia. Hum Brain Mapp 34:973–984. 10.1002/HBM.21484 22109837 10.1002/hbm.21484 PMC6870114

[18] Whitwell JL, Avula R, Senjem ML et al (2010) Gray and white matter water diffusion in the syndromic variants of frontotemporal dementia. Neurology 74:1279–1287. 10.1212/WNL.0B013E3181D9EDDE/SUPPL_FILE/TABLE_E-1.DOC 20404309 10.1212/WNL.0b013e3181d9edde PMC2860485

[19] Zhang Y, Schuff N, Du AT et al (2009) White matter damage in frontotemporal dementia and Alzheimer’s disease measured by diffusion MRI. Brain 132:2579–2592. 10.1093/BRAIN/AWP071 19439421 10.1093/brain/awp071 PMC2732263

[20] Hornberger M, Geng J, Hodges JR (2011) Convergent gray and white matter evidence of orbitofrontal cortex changes related to disinhibition in behavioral variant frontotemporal dementia. Brain 134:2502–2512. 10.1093/BRAIN/AWR173 21785117 10.1093/brain/awr173

[21] Jakabek D, Power BD, Macfarlane MD et al (2018) Regional structural hypo- and hyperconnectivity of frontal-striatal and frontal-thalamic pathways in behavioral variant frontotemporal dementia. Hum Brain Mapp 39:4083–4093. 10.1002/HBM.24233 29923666 10.1002/hbm.24233 PMC6866429

[22] Meijboom R, Steketee RME, Ham LS et al (2017) Differential hemispheric predilection of microstructural white matter and functional connectivity abnormalities between respectively semantic and behavioral variant frontotemporal dementia. J Alzheimers Dis 56:789–804. 10.3233/JAD-160564 28059782 10.3233/JAD-160564

[23] Routier A, Habert MO, Bertrand A et al (2018) Structural, microstructural, and metabolic alterations in primary progressive aphasia variants. Front Neurol 9:406523. 10.3389/FNEUR.2018.00766/BIBTEX 10.3389/fneur.2018.00766 PMC6153366 30279675

[24] Reyes PA, Rueda ADP, Uriza F, Matallana DL (2019) Networks disrupted in linguistic variants of frontotemporal dementia. Front Neurol. 10.3389/FNEUR.2019.00903 31507513 10.3389/fneur.2019.00903 PMC6716200

[25] Bouchard LO, Wilson MA, Laforce R, Duchesne S (2019) White matter damage in the semantic variant of primary progressive aphasia. Can J Neurol Sci 46:373–382. 10.1017/CJN.2019.37 31030675 10.1017/cjn.2019.37

[26] Brambati SM, Amici S, Racine CA et al (2015) Longitudinal gray matter contraction in three variants of primary progressive aphasia: a tenser-based morphometry study. Neuroimage Clin 8:345–355. 10.1016/J.NICL.2015.01.011 26106560 10.1016/j.nicl.2015.01.011 PMC4473099

[27] Catani M, Mesulam MM, Jakobsen E et al (2013) A novel frontal pathway underlies verbal fluency in primary progressive aphasia. Brain 136:2619–2628. 10.1093/BRAIN/AWT163 23820597 10.1093/brain/awt163 PMC3722349

[28] Santillo AF, Mårtensson J, Lindberg O et al (2013) Diffusion tensor tractography versus volumetric imaging in the diagnosis of behavioral variant frontotemporal dementia. PLoS ONE 8:e66932. 10.1371/JOURNAL.PONE.0066932 23874403 10.1371/journal.pone.0066932 PMC3715470

[29] McMillan CT, Brun C, Siddiqui S et al (2012) White matter imaging contributes to the multimodal diagnosis of frontotemporal lobar degeneration. Neurology 78:1761–1768. 10.1212/WNL.0B013E31825830BD/SUPPL_FILE/TABLE_E-2.DOCX 22592372 10.1212/WNL.0b013e31825830bd PMC3359585

[30] Cruz-Sanabria F, Reyes PA, Triviño-Martínez C et al (2021) Exploring signatures of neurodegeneration in early-onset older-age bipolar disorder and behavioral variant frontotemporal dementia. Front Neurol 12:713388. 10.3389/FNEUR.2021.713388/BIBTEX 34539558 10.3389/fneur.2021.713388 PMC8446277

[31] Rajagopalan V, Pioro EP (2021) Degeneration of gray and white matter differs between hypometabolic and hypermetabolic brain regions in a patient with ALS-FTD: a longitudinal MRI − PET multimodal study. Amyotroph Lateral Scler Frontotemporal Degener 22:127–132. 10.1080/21678421.2020.1818784 32924608 10.1080/21678421.2020.1818784

[32] Omer T, Finegan E, Hutchinson S et al (2017) Neuroimaging patterns along the ALS-FTD spectrum: a multiparametric imaging study. Amyotroph Lateral Scler Frontotemporal Degener 18:611–623. 10.1080/21678421.2017.1332077 28562080 10.1080/21678421.2017.1332077

[33] Lam BYK, Halliday GM, Irish M et al (2014) Longitudinal white matter changes in frontotemporal dementia subtypes. Hum Brain Mapp 35:3547–3557. 10.1002/HBM.22420 25050433 10.1002/hbm.22420 PMC6869363

[34] Tu S, Leyton CE, Hodges JR et al (2016) Divergent longitudinal propagation of white matter degradation in logopenic and semantic variants of primary progressive aphasia. J Alzheimers Dis 49:853–861. 10.3233/JAD-150626 26484929 10.3233/JAD-150626

[35] Yu J, Lee TMC (2019) The longitudinal decline of white matter microstructural integrity in behavioral variant frontotemporal dementia and its association with executive function. Neurobiol Aging 76:62–70. 10.1016/J.NEUROBIOLAGING.2018.12.005 30703627 10.1016/j.neurobiolaging.2018.12.005

[36] Mahoney CJ, Simpson IJA, Nicholas JM et al (2015) Longitudinal diffusion tensor imaging in frontotemporal dementia. Ann Neurol 77:33–46. 10.1002/ANA.24296 25363208 10.1002/ana.24296 PMC4305215

[37] Kassubek J, Müller HP, Del TK et al (2018) Longitudinal diffusion tensor imaging resembles patterns of pathology progression in behavioral variant frontotemporal dementia (bvFTD). Front Aging Neurosci 10:302570. 10.3389/FNAGI.2018.00047/BIBTEX 10.3389/fnagi.2018.00047 PMC5845670 29559904

[38] Staffaroni AM, Ljubenkov PA, Kornak J et al (2019) Longitudinal multimodal imaging and clinical endpoints for frontotemporal dementia clinical trials. Brain 142:443–459. 10.1093/BRAIN/AWY319 30698757 10.1093/brain/awy319 PMC6351779

[39] Elahi FM, Marx G, Cobigo Y et al (2017) Longitudinal white matter change in frontotemporal dementia subtypes and sporadic late onset Alzheimer’s disease. Neuroimage Clin 16:595–603. 10.1016/J.NICL.2017.09.007 28975068 10.1016/j.nicl.2017.09.007 PMC5614750

[40] Floeter MK, Bageac D, Danielian LE et al (2016) Longitudinal imaging in C9orf72 mutation carriers: relationship to phenotype. Neuroimage Clin 12:1035–1043. 10.1016/J.NICL.2016.10.014 27995069 10.1016/j.nicl.2016.10.014 PMC5153604

[41] Agosta F, Galantucci S, Magnani G et al (2015) MRI signatures of the frontotemporal lobar degeneration continuum. Hum Brain Mapp 36:2602–2614. 10.1002/HBM.22794 25821176 10.1002/hbm.22794 PMC6869605

[42] Mahoney CJ, Malone IB, Ridgway GR et al (2013) White matter tract signatures of the progressive aphasias. Neurobiol Aging 34:1687–1699. 10.1016/J.NEUROBIOLAGING.2012.12.002 23312804 10.1016/j.neurobiolaging.2012.12.002 PMC3601331

[43] Tee BL, Gorno-Tempini ML (2019) Primary progressive aphasia: a model for neurodegenerative disease. Curr Opin Neurol 32:255–265. 10.1097/WCO.0000000000000673 30694922 10.1097/WCO.0000000000000673 PMC6602793

[44] Warren JD, Rohrer JD, Schott JM et al (2013) Molecular nexopathies: a new paradigm of neurodegenerative disease. Trends Neurosci 36:561–569. 10.1016/J.TINS.2013.06.007 23876425 10.1016/j.tins.2013.06.007 PMC3794159

[45] Belder CRS, Marshall CR, Jiang J et al (2023) Primary progressive aphasia: six questions in search of an answer. J Neurol 271:1028–1046. 10.1007/S00415-023-12030-4 37906327 10.1007/s00415-023-12030-4 PMC10827918

[46] Franzmeier N, Neitzel J, Rubinski A et al (2020) Functional brain architecture is associated with the rate of tau accumulation in Alzheimer’s disease. Nat Commun 11:347. 10.1038/s41467-019-14159-1 31953405 10.1038/s41467-019-14159-1 PMC6969065

[47] Franzmeier N, Brendel M, Beyer L et al (2022) Tau deposition patterns are associated with functional connectivity in primary tauopathies. Nat Commun 13:1362. 10.1038/s41467-022-28896-3 35292638 10.1038/s41467-022-28896-3 PMC8924216

[48] Schulthess I, Gorges M, Müller HP et al (2016) Functional connectivity changes resemble patterns of pTDP-43 pathology in amyotrophic lateral sclerosis. Sci Rep 6:38391. 10.1038/srep38391 27929102 10.1038/srep38391 PMC5144012

[49] Buckner RL, Sepulcre J, Talukdar T et al (2009) Cortical hubs revealed by intrinsic functional connectivity: mapping, assessment of stability, and relation to Alzheimer’s disease. J Neurosci 29:1860–1873. 10.1523/JNEUROSCI.5062-08.2009 19211893 10.1523/JNEUROSCI.5062-08.2009 PMC2750039

[50] Seeley WW, Crawford RK, Zhou J et al (2009) Neurodegenerative diseases target large-scale human brain networks. Neuron 62:42–52. 10.1016/j.neuron.2009.03.024 19376066 10.1016/j.neuron.2009.03.024 PMC2691647

[51] Frost B, Diamond MI (2009) Prion-like mechanisms in neurodegenerative diseases. Nat Rev Neurosci 11:155–159. 10.1038/nrn2786 20029438 10.1038/nrn2786 PMC3648341

[52] Müller HP, Turner MR, Grosskreutz J et al (2016) A large-scale multicentre cerebral diffusion tensor imaging study in amyotrophic lateral sclerosis. J Neurol Neurosurg Psychiatry 87:570–579. 10.1136/JNNP-2015-311952 26746186 10.1136/jnnp-2015-311952

[53] Kalra S, Müller HP, Ishaque A et al (2020) A prospective harmonized multicenter DTI study of cerebral white matter degeneration in ALS. Neurology 95:E943–E952. 10.1212/WNL.0000000000010235 32646955 10.1212/WNL.0000000000010235 PMC7668555

[54] Huppertz HJ, Kröll-Seger J, Klöppel S et al (2010) Intra- and interscanner variability of automated voxel-based volumetry based on a 3D probabilistic atlas of human cerebral structures. Neuroimage 49:2216–2224. 10.1016/J.NEUROIMAGE.2009.10.066 19878722 10.1016/j.neuroimage.2009.10.066

[55] Opfer R, Suppa P, Kepp T et al (2016) Atlas based brain volumetry: How to distinguish regional volume changes due to biological or physiological effects from inherent noise of the methodology. Magn Reson Imaging 34:455–461. 10.1016/J.MRI.2015.12.031 26723849 10.1016/j.mri.2015.12.031

[56] Höglinger GU, Huppertz HJ, Wagenpfeil S et al (2014) Tideglusib reduces progression of brain atrophy in progressive supranuclear palsy in a randomized trial. Mov Disord 29:479–487. 10.1002/MDS.25815 24488721 10.1002/mds.25815

[57] Frings L, Mader I, Landwehrmeyer BG et al (2012) Quantifying change in individual subjects affected by frontotemporal lobar degeneration using automated longitudinal MRI volumetry. Hum Brain Mapp 33:1526–1535. 10.1002/HBM.21304 21618662 10.1002/hbm.21304 PMC6869947

[58] Kassubek J, Pinkhardt EH, Dietmaier A et al (2011) Fully automated atlas-based MR imaging volumetry in Huntington disease, compared with manual volumetry. Am J Neuroradiol 32:1328–1332. 10.3174/AJNR.A2514 21680653 10.3174/ajnr.A2514 PMC7966058

[59] Wiesenfarth M, Huppertz HJ, Dorst J et al (2023) Structural and microstructural neuroimaging signature of C9orf72-associated ALS: a multiparametric MRI study. Neuroimage Clin 39:103505. 10.1016/J.NICL.2023.103505 37696099 10.1016/j.nicl.2023.103505 PMC10500452

[60] Jessen F, Amariglio RE, Van Boxtel M et al (2014) A conceptual framework for research on subjective cognitive decline in preclinical Alzheimer’s disease. Alzheimers Dement 10:844–852. 10.1016/J.JALZ.2014.01.001 24798886 10.1016/j.jalz.2014.01.001 PMC4317324

[61] Neary D, Snowden JS, Gustafson L et al (1998) Frontotemporal lobar degeneration: a consensus on clinical diagnostic criteria. Neurology 51:1546–1554. 10.1212/WNL.51.6.1546 9855500 10.1212/wnl.51.6.1546

[62] Miyagawa T, Brushaber D, Syrjanen J et al (2020) Use of the CDR® plus NACC FTLD in mild FTLD: data from the ARTFL/LEFFTDS consortium. Alzheimers Dement 16:79–90. 10.1016/J.JALZ.2019.05.013 31477517 10.1016/j.jalz.2019.05.013 PMC6949373

[63] Mueller HP, Unrath A, Sperfeld AD et al (2007) Diffusion tensor imaging and tractwise fractional anisotropy statistics: quantitative analysis in white matter pathology. Biomed Eng Online 6:1–10. 10.1186/1475-925X-6-42/TABLES/4 17996104 10.1186/1475-925X-6-42 PMC2186341

[64] Müller HP, Grön G, Sprengelmeyer R et al (2013) Evaluating multicenter DTI data in Huntington’s disease on site specific effects: an ex post facto approach. Neuroimage Clin 2:161–167. 10.1016/J.NICL.2012.12.005 24179771 10.1016/j.nicl.2012.12.005 PMC3777841

[65] Menke RAL, Körner S, Filippini N et al (2014) Widespread gray matter pathology dominates the longitudinal cerebral MRI and clinical landscape of amyotrophic lateral sclerosis. Brain 137:2546–2555. 10.1093/BRAIN/AWU162 24951638 10.1093/brain/awu162 PMC4132644

[66] Brett M, Johnsrude IS, Owen AM (2002) The problem of functional localization in the human brain. Nat Rev Neurosci 3:243–249. 10.1038/nrn756 11994756 10.1038/nrn756

[67] Smith SM, Jenkinson M, Johansen-Berg H et al (2006) Tract-based spatial statistics: voxelwise analysis of multi-subject diffusion data. Neuroimage 31:1487–1505. 10.1016/J.NEUROIMAGE.2006.02.024 16624579 10.1016/j.neuroimage.2006.02.024

[68] Müller HP, Kassubek J (2013) Diffusion tensor magnetic resonance imaging in the analysis of neurodegenerative diseases. J Vis Exp. 10.3791/50427 23928996 10.3791/50427 PMC3846464

[69] Müller HP, Unrath A, Riecker A et al (2009) Intersubject variability in the analysis of diffusion tensor images at the group level: fractional anisotropy mapping and fiber tracking techniques. Magn Reson Imaging 27:324–334. 10.1016/J.MRI.2008.07.003 18701228 10.1016/j.mri.2008.07.003

[70] Rosskopf J, Müller HP, Dreyhaupt J et al (2015) Ex post facto assessment of diffusion tensor imaging metrics from different MRI protocols: preparing for multicentre studies in ALS. Amyotroph Lateral Scler Frontotemporal Degener 16:92–101. 10.3109/21678421.2014.977297 25574564 10.3109/21678421.2014.977297

[71] Mesulam MM, Coventry C, Bigio EH et al (2021) Nosology of primary progressive aphasia and the neuropathology of language. Adv Exp Med Biol 1281:33–49. 10.1007/978-3-030-51140-1_3 33433867 10.1007/978-3-030-51140-1_3 PMC8103786

[72] Agosta F, Scola E, Canu E et al (2012) White matter damage in frontotemporal lobar degeneration spectrum. Cereb Cortex 22:2705–2714. 10.1093/CERCOR/BHR288 21988828 10.1093/cercor/bhr288

[73] D’Anna L, Mesulam MM, Thiebaut De Schotten M et al (2016) Frontotemporal networks and behavioral symptoms in primary progressive aphasia. Neurology 86:1393–1399. 10.1212/WNL.0000000000002579/SUPPL_FILE/FIGURE_E-4.JPEG 26992858 10.1212/WNL.0000000000002579 PMC4831038

[74] Acosta-Cabronero J, Patterson K, Fryer TD et al (2011) Atrophy, hypometabolism and white matter abnormalities in semantic dementia tell a coherent story. Brain 134:2025–2035. 10.1093/BRAIN/AWR119 21646331 10.1093/brain/awr119

[75] Jiskoot LC, Panman JL, Meeter LH et al (2019) Longitudinal multimodal MRI as prognostic and diagnostic biomarker in presymptomatic familial frontotemporal dementia. Brain 142:193–208. 10.1093/BRAIN/AWY288 30508042 10.1093/brain/awy288 PMC6308313

[76] Kassubek J, Müller HP, Del Tredici K et al (2014) Diffusion tensor imaging analysis of sequential spreading of disease in amyotrophic lateral sclerosis confirms patterns of TDP-43 pathology. Brain 137:1733–1740. 10.1093/BRAIN/AWU090 24736303 10.1093/brain/awu090

[77] Kassubek J, Müller HP, Del Tredici K et al (2018) Imaging the pathoanatomy of amyotrophic lateral sclerosis in vivo: targeting a propagation-based biological marker. J Neurol Neurosurg Psychiatry 89:374–381. 10.1136/JNNP-2017-316365 29101254 10.1136/jnnp-2017-316365 PMC5869447

[78] Huppertz HJ, Möller L, Südmeyer M et al (2016) Differentiation of neurodegenerative parkinsonian syndromes by volumetric magnetic resonance imaging analysis and support vector machine classification. Mov Disord 31:1506–1517. 10.1002/MDS.26715 27452874 10.1002/mds.26715

[79] Müller HP, Unrath A, Huppertz HJ et al (2012) Neuroanatomical patterns of cerebral white matter involvement in different motor neuron diseases as studied by diffusion tensor imaging analysis. Amyotroph Lateral Scler 13:254–264. 10.3109/17482968.2011.653571 22409361 10.3109/17482968.2011.653571

[80] Kunimatsu A, Aoki S, Masutani Y et al (2004) The optimal trackability threshold of fractional anisotropy for diffusion tensor tractography of the corticospinal tract. Magn Reson Med Sci 3:11–17. 10.2463/MRMS.3.11 16093615 10.2463/mrms.3.11

[81] Genovese CR, Lazar NA, Nichols T (2002) Thresholding of statistical maps in functional neuroimaging using the false discovery rate. Neuroimage 15:870–878. 10.1006/NIMG.2001.1037 11906227 10.1006/nimg.2001.1037

[82] Behler A, Kassubek J, Müller HP (2021) Age-related alterations in DTI metrics in the human brain—consequences for age correction. Front Aging Neurosci 13:682109. 10.3389/FNAGI.2021.682109/BIBTEX 34211389 10.3389/fnagi.2021.682109 PMC8239142

[83] Breiman L (2001) Random forests. Mach Learn 45:5–32. 10.1023/A:1010933404324/METRICS

[84] Lecun Y, Bengio Y, Hinton G (2015) Deep learning. Nature 521(7553):436–444. 10.1038/nature14539 26017442 10.1038/nature14539

[85] Janiesch C, Zschech P, Heinrich K (2021) Machine learning and deep learning. Electron Mark 31:685–695. 10.1007/s12525-021-00475-2

[86] Volkmann H, Höglinger GU, Grön G et al (2025) MRI classification of progressive supranuclear palsy, Parkinson disease and controls using deep learning and machine learning algorithms for the identification of regions and tracts of interest as potential biomarkers. Comput Biol Med 185:109518. 10.1016/J.COMPBIOMED.2024.109518 39662313 10.1016/j.compbiomed.2024.109518

[87] Wong TT (2015) Performance evaluation of classification algorithms by k-fold and leave-one-out cross validation. Pattern Recognit 48:2839–2846. 10.1016/J.PATCOG.2015.03.009

[88] Pedregosa F, Varoquaux G, Gramfort A et al (2012) Scikit-learn: machine learning in python. J Mach Learn Res 12:2825–2830

[89] Agosta F, Galantucci S, Canu E et al (2013) Disruption of structural connectivity along the dorsal and ventral language pathways in patients with nonfluent and semantic variant primary progressive aphasia: a DT MRI study and a literature review. Brain Lang 127:157–166. 10.1016/J.BANDL.2013.06.003 23890877 10.1016/j.bandl.2013.06.003

[90] Mandelli ML, Caverzasi E, Binney RJ et al (2014) Frontal white matter tracts sustaining speech production in primary progressive aphasia. J Neurosci 34:9754–9767. 10.1523/JNEUROSCI.3464-13.2014 25031413 10.1523/JNEUROSCI.3464-13.2014 PMC4099550

[91] Tafuri B, Filardi M, Urso D et al (2023) Asymmetry of radiomics features in the white matter of patients with primary progressive aphasia. Front Aging Neurosci 15:1120935. 10.3389/FNAGI.2023.1120935 37213534 10.3389/fnagi.2023.1120935 PMC10196268

[92] Lombardi J, Mayer B, Semler E et al (2021) Quantifying progression in primary progressive aphasia with structural neuroimaging. Alzheimer’s & Dementia 17:1595–1609. 10.1002/ALZ.12323 10.1002/alz.12323 33787063

[93] Albrecht F, Bisenius S, Schaack RM et al (2017) Disentangling the neural correlates of corticobasal syndrome and corticobasal degeneration with systematic and quantitative ALE meta-analyses. npj Parkinson’s Dis 3:12. 10.1038/s41531-017-0012-6 28649612 10.1038/s41531-017-0012-6 PMC5459811

[94] Boxer AL, Geschwind MD, Belfor N et al (2006) Patterns of brain atrophy that differentiate corticobasal degeneration syndrome from progressive supranuclear palsy. Arch Neurol 63:81–86. 10.1001/ARCHNEUR.63.1.81 16401739 10.1001/archneur.63.1.81

[95] Whitwell JL, Höglinger GU, Antonini A et al (2017) Radiological biomarkers for diagnosis in PSP: where are we and where do we need to be? Mov Disord 32:955. 10.1002/MDS.27038 28500751 10.1002/mds.27038 PMC5511762

[96] Spinelli EG, Mandelli ML, Miller ZA et al (2017) Typical and atypical pathology in primary progressive aphasia variants. Ann Neurol 81:430–443. 10.1002/ANA.24885 28133816 10.1002/ana.24885 PMC5421819

[97] Bocchetta M, Iglesias JE, Russell LL et al (2019) Segmentation of medial temporal subregions reveals early right-sided involvement in semantic variant PPA. Alzheimers Res Ther 11:1–9. 10.1186/S13195-019-0489-9/TABLES/1 31077248 10.1186/s13195-019-0489-9 PMC6511178

[98] Lampe L, Huppertz HJ, Anderl-Straub S et al (2023) Multiclass prediction of different dementia syndromes based on multi-centric volumetric MRI imaging. Neuroimage Clin 37:103320. 10.1016/J.NICL.2023.103320 36623349 10.1016/j.nicl.2023.103320 PMC9850041

[99] Maller JJ, Welton T, Middione M et al (2019) Revealing the hippocampal connectome through super-resolution 1150-direction diffusion MRI. Sci Rep 9:2418. 10.1038/s41598-018-37905-9 30787303 10.1038/s41598-018-37905-9 PMC6382767

[100] Von Der Heide RJ, Skipper LM, Klobusicky E, Olson IR (2013) Dissecting the uncinate fasciculus: disorders, controversies and a hypothesis. Brain 136:1692. 10.1093/BRAIN/AWT094 23649697 10.1093/brain/awt094 PMC3673595

[101] Goetschius LG, Hein TC, Mattson WI et al (2019) Amygdala-prefrontal cortex white matter tracts are widespread, variable and implicated in amygdala modulation in adolescents. Neuroimage 191:278. 10.1016/J.NEUROIMAGE.2019.02.009 30790672 10.1016/j.neuroimage.2019.02.009 PMC6440813

[102] Nigro S, Tafuri B, Urso D et al (2021) Altered structural brain networks in linguistic variants of frontotemporal dementia. Brain Imaging Behav 16:1113. 10.1007/S11682-021-00560-2 34755293 10.1007/s11682-021-00560-2 PMC9107413

[103] Shapiro NL, Todd EG, Billot B et al (2022) In vivo hypothalamic regional volumetry across the frontotemporal dementia spectrum. Neuroimage Clin 35:103084. 10.1016/J.NICL.2022.103084 35717886 10.1016/j.nicl.2022.103084 PMC9218583

[104] Brambati SM, Rankin KP, Narvid J et al (2009) Atrophy progression in semantic dementia with asymmetric temporal involvement: a tensor-based morphometry study. Neurobiol Aging 30:103–111. 10.1016/J.NEUROBIOLAGING.2007.05.014 17604879 10.1016/j.neurobiolaging.2007.05.014 PMC2643844

[105] Tetzloff KA, Duffy JR, Clark HM et al (2018) Longitudinal structural and molecular neuroimaging in agrammatic primary progressive aphasia. Brain 141:302–317. 10.1093/BRAIN/AWX293 29228180 10.1093/brain/awx293 PMC5837339

[106] Caso F, Mandelli ML, Henry M et al (2014) In vivo signatures of nonfluent/agrammatic primary progressive aphasia caused by FTLD pathology. Neurology 82:239–247. 10.1212/WNL.0000000000000031 24353332 10.1212/WNL.0000000000000031 PMC3902758

[107] Rohrer JD, Geser F, Zhou J et al (2010) TDP-43 subtypes are associated with distinct atrophy patterns in frontotemporal dementia. Neurology 75:2204–2211. 10.1212/WNL.0B013E318202038C/SUPPL_FILE/TABLE_E-1.DOCX 21172843 10.1212/WNL.0b013e318202038c PMC3013589

[108] Logroscino G, Piccininni M, Graff C et al (2023) Incidence of syndromes associated with frontotemporal lobar degeneration in 9 European countries. JAMA Neurol 80:279–286. 10.1001/JAMANEUROL.2022.5128 36716024 10.1001/jamaneurol.2022.5128 PMC9887528

[109] Müller HP, Kassubek J (2024) Toward diffusion tensor imaging as a biomarker in neurodegenerative diseases: technical considerations to optimize recordings and data processing. Front Hum Neurosci 18:1378896. 10.3389/FNHUM.2024.1378896/BIBTEX 38628970 10.3389/fnhum.2024.1378896 PMC11018884

[110] Grossman M (2012) The non-fluent/agrammatic variant of primary progressive aphasia. Lancet Neurol 11:545–555. 10.1016/S1474-4422(12)70099-6 22608668 10.1016/S1474-4422(12)70099-6 PMC3361730

[111] Josephs KA, Duffy JR, Strand EA et al (2013) Syndromes dominated by apraxia of speech show distinct characteristics from agrammatic PPA. Neurology 81:337. 10.1212/WNL.0B013E31829C5ED5 23803320 10.1212/WNL.0b013e31829c5ed5 PMC3772832

[112] Josephs KA, Duffy JR, Strand EA et al (2012) Characterizing a neurodegenerative syndrome: primary progressive apraxia of speech. Brain 135:1522–1536. 10.1093/BRAIN/AWS032 22382356 10.1093/brain/aws032 PMC3338923

[113] Meade G, Whitwell JL, Dickson DW et al (2024) Primary progressive apraxia of speech caused by TDP-43. Neurol Genet. 10.1212/NXG.0000000000200134/SUPPL_FILE/SUPPLEMENTARY_TABLE1.PDF 38515991 10.1212/NXG.0000000000200134 PMC10955458

[114] Harris JM, Gall C, Thompson JC et al (2013) Classification and pathology of primary progressive aphasia. Neurology 81:1832–1839. 10.1212/01.WNL.0000436070.28137.7B 24142474 10.1212/01.wnl.0000436070.28137.7b

[115] Tetzloff KA, Duffy JR, Clark HM et al (2019) Progressive agrammatic aphasia without apraxia of speech as a distinct syndrome. Brain 142:2466–2482. 10.1093/BRAIN/AWZ157 31199471 10.1093/brain/awz157 PMC6658844

[116] Erkoyun HU, Groot C, Heilbron R et al (2020) A clinical-radiological framework of the right temporal variant of frontotemporal dementia. Brain 143:2831. 10.1093/BRAIN/AWAA225 32830218 10.1093/brain/awaa225 PMC9172625

[117] Mackenzie IRA, Neumann M (2016) Molecular neuropathology of frontotemporal dementia: insights into disease mechanisms from postmortem studies. J Neurochem 138:54–70. 10.1111/JNC.13588 27306735 10.1111/jnc.13588

[118] Ducharme S, Pijnenburg Y, Rohrer JD et al (2024) Identifying and diagnosing TDP-43 neurodegenerative diseases in psychiatry. Am J Geriatr Psychiatry 32:98–113. 10.1016/J.JAGP.2023.08.017 37741764 10.1016/j.jagp.2023.08.017 PMC11270911

[119] Santangelo R, Coppi E, Ferrari L et al (2014) Cerebrospinal fluid biomarkers can play a pivotal role in the diagnostic work up of primary progressive aphasia. J Alzheimers Dis 43:1429–1440. 10.3233/JAD-141122 10.3233/JAD-141122 25201781

