# Supplementary material for: Multiparametric MRI-based biomarkers in the non-fluent and semantic variants of primary progressive aphasia
Source: J Neurol. 2025 Jul 2;272(8):490. doi: 10.1007/s00415-025-13215-9 (PMC12222332; doi:10.1007/s00415-025-13215-9)
Supplement: Supplementary file 1 — Supplementary file1 (DOCX 147 KB) [file 415_2025_13215_MOESM1_ESM.docx]

**Multiparametric MRI-Based Biomarkers in the non-fluent and semantic variants of primary progressive aphasia**

Marco Michelutti^1,2,3^, MD, Hans-Jürgen Huppertz^4^, MD, Heiko Volkmann^2^, MS, Sarah Anderl-Straub^2^, PhD, Daniele Urso^3^, MD, MPH, Benedetta Tafuri^3,5^, PhD, Salvatore Nigro^3,6^, PhD, Paolo Manganotti^1^, MD, PhD, Leonie Werner^2^, PhD, Jolina Lombardi^2^, PhD, Markus Otto^7^, MD, Giancarlo Logroscino^3^, MD, PhD, Hans-Peter Müller^2,8,9^, PhD*, Jan Kassubek^2^, MD*

*shared senior authorship

^1^ Neurology Unit, Department of Medical, Surgical and Health Sciences, University of Trieste, Trieste, Italy.

^2^ Department of Neurology, University Hospital Ulm, Ulm, Germany

^3^ Center for Neurodegenerative Diseases and the Aging Brain, University of Bari Aldo Moro at Pia Fondazione "Card. G. Panico", Tricase, Italy.

^4^ Swiss Epilepsy Clinic, Klinik Lengg, Zürich, Switzerland

^5^ Department of Engineering of Innovation, University of Salento, Lecce, Italy

^6^ Institute of Nanotechnology, National Research Council (CNR-NANOTEC) c/o Campus Ecotekne, via Monteroni, 73100 Lecce, Italy

^7^ Department of Neurology, University Hospital Halle, Martin Luther University, Halle (Saale), Germany

^8^ Department of Diagnostic and Interventional Radiology, University Hospital Ulm, Ulm, Germany

^9^ Department of Nuclear Medicine, University Hospital Ulm, Ulm, Germany

**Corresponding Author**:

Marco Michelutti, MD

Neurology Unit, Department of Medical, Surgical and Health Sciences, University of Trieste, Trieste, Italy.

Strada di Fiume 447, 34149 Trieste (TS)

Email: MARCO.MICHELUTTI@studenti.units.it

ORCID: 0009-0009-4308-8456

**Supplementary Table 1** Whole-brain-based spatial statistics for cross-sectional comparison of FA maps of patients with nfPPA with available longitudinal data (N=10) vs controls (N=39), at baseline.

| **Anatomical location** | **L/R** | **X** | **Y** | **Z** | **N. of voxels** |
| --- | --- | --- | --- | --- | --- |
| Corpus callosum | L/R | ±7 | 14 | 20 | 48205 |
| Left temporal lobe | L | 41 | -15 | -10 | 6966 |

This table shows details of the clusters where FA changes were detected in the subgroup of nfPPA patients with available longitudinal data (n=10) compared to controls (N=39) by whole-brain based spatial statistics, at baseline (**Figure 2**).

**Legend** FA Fractional anisotropy**;** nfPPA: non fluent primary progressive aphasia; L/R: left/right.

**Supplementary Table 2** Whole-brain-based spatial statistics for cross-sectional comparison of FA maps of patients with nfPPA with available longitudinal data (N=10) vs controls (N=39), at 1 year follow-up.

| **Anatomical location** | **L/R** | **X** | **Y** | **Z** | **N. of voxels** |
| --- | --- | --- | --- | --- | --- |
| Corpus callosum | L/R | ±12 | 26 | 41 | 64861 |

This table shows details of the clusters where FA changes were detected in the subgroup of nfPPA patients with available longitudinal data (n=10) compared to controls (N=39) by whole-brain based spatial statistics, at 1 year follow-up (**Figure 2**).

**Legend** FA Fractional anisotropy**;** nfPPA: non fluent primary progressive aphasia; L/R: left/right.

**Supplementary Table 3** Whole-brain-based spatial statistics for cross-sectional comparison of FA maps of patients with svPPA with available longitudinal data (N=6) vs controls (N=39), at baseline.

C

| **Anatomical location** | **L/R** | **X** | **Y** | **Z** | **N. of voxels** |
| --- | --- | --- | --- | --- | --- |
| Temporal lobe | L | -52 | -43 | -5 | 7054 |
| Basal ganglia | R | 33 | -8 | 8 | 3070 |
| Basal ganglia | L | -42 | -16 | -9 | 6338 |

This table shows details of the clusters where FA changes were detected in the subgroup of svPPA patients with available longitudinal data (n=6) compared to controls (N=39) by whole-brain based spatial statistics, at baseline (**Figure 3**).

**Legend** FA Fractional anisotropy**;** svPPA: semantic variant of primary progressive aphasia; L/R: left/right.

**Supplementary Table 4** Whole-brain-based spatial statistics for cross-sectional comparison of FA maps of patients with svPPA with available longitudinal data (N=6) vs controls (N=39), at 1 year follow-up.

| **Anatomical location** | **L/R** | **X** | **Y** | **Z** | **N. of voxels** |
| --- | --- | --- | --- | --- | --- |
| Temporal lobe | L | -52 | -43 | -5 | 9841 |
| Basal ganglia | R | 29 | -10 | 5 | 2265 |

This table shows details of the clusters where FA changes were detected in the subgroup of svPPA patients with available longitudinal data (n=6) compared to controls (N=39) by whole-brain based spatial statistics, at 1 year follow-up (**figure 3**).

**Legend** FA Fractional anisotropy**;** svPPA: semantic variant of primary progressive aphasia; L/R: left/right.

**Supplementary Table 5** Longitudinal FA differences in the SOIs at group-level for patients with nfPPA.

| **ABV analysis** | **Controls** | | **nfPPA (baseline)** | | **nfPPA (FU)** | | **z-scores** | | **Group Comparison** | |
| --- | --- | --- | --- | --- | --- | --- | --- | --- | --- | --- |
|  | Mean | SD | Mean | SD | Mean | SD | nfPPA (baseline) vs Controls | nfPPA (FU) vs Controls | nfPPA (baseline) vs Controls | nfPPA (FU) vs Controls |
| Cerebrum GM | 533.7 | 55.0 | 448.9 | 26.6 | 438.0 | 22.2 | -1.2 | -1.4 | <0.001 | <0.001 |
| Cerebrum WM | 389.5 | 28.8 | 368.5 | 43.8 | 355.7 | 37.3 | -0.2 | -0.4 | ns | ns |
| Frontal lobe R | 151.6 | 10.8 | 128.5 | 9.5 | 123.5 | 11.5 | -1.2 | -1.6 | 0.004 | 0.001 |
| Frontal lobe L | 153.0 | 11.3 | 127.4 | 13.7 | 121.6 | 15.2 | -1.4 | -1.8 | 0.008 | 0.003 |
| Temporal lobe R | 92.3 | 6.1 | 86.2 | 7.6 | 84.9 | 7.3 | -0.3 | -0.4 | ns | ns |
| Temporal lobe L | 93.7 | 6.7 | 83.7 | 6.2 | 81.4 | 5.8 | -0.8 | -1.1 | ns | 0.007 |
| Parietal lobe R | 82.8 | 5.4 | 74.9 | 4.6 | 72.9 | 4.2 | -0.6 | -0.9 | ns | 0.006 |
| Parietal lobe L | 86.1 | 6.2 | 76.5 | 5.8 | 73.7 | 5.2 | -0.8 | -1.2 | ns | 0.002 |
| Occipital lobe R | 62.1 | 4.7 | 57.2 | 6.6 | 56.3 | 6.4 | -0.4 | -0.6 | ns | ns |
| Occipital lobe L | 60.0 | 4.9 | 54.0 | 5.0 | 53.1 | 5.0 | -0.6 | -0.8 | ns | ns |
| Insula R | 8.1 | 0.8 | 7.4 | 0.6 | 7.3 | 0.6 | -0.4 | -0.7 | ns | ns |
| Insula L | 8.4 | 0.7 | 7.6 | 0.6 | 7.4 | 0.6 | -0.6 | -1.0 | ns | 0.025 |
| Cerebellum | 113.3 | 10.4 | 105.6 | 10.2 | 104.2 | 8.3 | -0.3 | -0.5 | ns | ns |
| Brainstem | 30.1 | 2.5 | 28.3 | 3.2 | 27.6 | 3.0 | -0.2 | -0.5 | ns | ns |
| Hippocampus R | 3.4 | 0.3 | 3.0 | 0.4 | 3.0 | 0.3 | -0.9 | -0.8 | ns | 0.023 |
| Hippocampus L | 3.2 | 0.3 | 2.8 | 0.3 | 2.8 | 0.3 | -1.1 | -1.3 | ns | ns |
| Amygdala R | 1.9 | 0.2 | 1.7 | 0.2 | 1.7 | 0.2 | -0.7 | -0.8 | ns | ns |
| Amygdala L | 1.7 | 0.2 | 1.5 | 0.2 | 1.5 | 0.2 | -1.1 | -1.3 | ns | 0.025 |
| Caudate R | 2.2 | 0.2 | 1.8 | 0.4 | 1.8 | 0.4 | -0.9 | -1.0 | ns | ns |
| Caudate L | 2.3 | 0.3 | 1.8 | 0.5 | 1.8 | 0.5 | -1.4 | -1.5 | ns | ns |
| Putamen R | 3.2 | 0.5 | 2.7 | 0.3 | 2.5 | 0.4 | -0.8 | -1.1 | 0.012 | 0.007 |
| Putamen L | 3.3 | 0.5 | 2.5 | 0.5 | 2.4 | 0.6 | -1.2 | -1.5 | 0.01 | 0.007 |
| Thalamus R | 6.0 | 0.6 | 5.4 | 0.4 | 5.2 | 0.4 | -0.6 | -0.8 | ns | 0.013 |
| Thalamus L | 6.3 | 0.7 | 5.4 | 0.4 | 5.3 | 0.4 | -0.9 | -1.2 | 0.006 | 0.001 |

This table shows the differences in SOI volumes (ml) at baseline between 10 patients with nfPPA at baseline and at one-year follow up versus controls (n=39). Mean and standard deviation are reported for each group. Z-scores were calculated as the difference between the subject's mean and the control group's mean, divided by the control group's standard deviation. FDR-corrected *p* values are reported if significant.

**Legend**: SOIs: Structures of Interest; ABV: Atlas-based-Volumetry; nfPPA: non-fluent variant of primary progressive aphasia; FU: follow-up; SD: standard deviation; GM: gray matter; WM: white matter; ns: non significant.

**Supplementary Table 6** Longitudinal FA differences in the SOIs at group-level for patients with svPPA.

| **ABV analysis** | **Controls** | | **svPPA (baseline)** | | **svPPA (FU)** | | **z-scores** | | **Group Comparison** | |
| --- | --- | --- | --- | --- | --- | --- | --- | --- | --- | --- |
|  | Mean | SD | Mean | SD | Mean | SD | svPPA (baseline) vs Controls | svPPA (FU) vs Controls | svPPA (baseline) vs Controls | svPPA (FU) vs Controls |
| Cerebrum GM | 533.7 | 55.0 | 455.9 | 56.5 | 435.8 | 57.4 | -1.0 | -1.4 | ns | ns |
| Cerebrum WM | 389.5 | 28.8 | 411.2 | 34.1 | 403.8 | 37.7 | 0.7 | 0.6 | ns | ns |
| Frontal lobe R | 151.6 | 10.8 | 150.0 | 9.8 | 145.6 | 10.9 | 0.4 | 0.0 | ns | ns |
| Frontal lobe L | 153.0 | 11.3 | 140.7 | 13.9 | 135.3 | 15.3 | -0.4 | -0.8 | ns | ns |
| Temporal lobe R | 92.3 | 6.1 | 86.9 | 10.2 | 82.4 | 9.8 | -0.2 | -0.7 | ns | ns |
| Temporal lobe L | 93.7 | 6.7 | 71.1 | 8.6 | 66.4 | 8.3 | -2.4 | -3.0 | 0.021 | 0.004 |
| Parietal lobe R | 82.8 | 5.4 | 83.4 | 8.8 | 81.8 | 9.6 | 0.5 | 0.3 | ns | ns |
| Parietal lobe L | 86.1 | 6.2 | 79.7 | 8.1 | 77.6 | 9.0 | -0.4 | -0.7 | ns | ns |
| Occipital lobe R | 62.1 | 4.7 | 62.7 | 7.3 | 61.9 | 6.8 | 0.6 | 0.5 | ns | ns |
| Occipital lobe L | 60.0 | 4.9 | 55.8 | 6.5 | 55.0 | 5.9 | -0.3 | -0.5 | ns | ns |
| Insula R | 8.1 | 0.8 | 7.5 | 0.8 | 7.1 | 0.8 | -0.3 | -0.9 | ns | ns |
| Insula L | 8.4 | 0.7 | 6.1 | 0.4 | 5.7 | 0.4 | -2.8 | -3.4 | <0.001 | <0.001 |
| Cerebellum | 113.3 | 10.4 | 118.3 | 6.2 | 115.2 | 6.3 | 1.0 | 0.6 | ns | ns |
| Brainstem | 30.1 | 2.5 | 32.1 | 2.4 | 31.5 | 2.4 | 1.0 | 0.9 | ns | ns |
| Hippocampus R | 3.4 | 0.3 | 3.1 | 0.5 | 2.9 | 0.5 | -0.5 | -1.2 | ns | ns |
| Hippocampus L | 3.2 | 0.3 | 2.2 | 0.4 | 2.1 | 0.5 | -3.5 | -4.0 | 0.023 | 0.016 |
| Amygdala R | 1.9 | 0.2 | 1.6 | 0.2 | 1.5 | 0.2 | -1.4 | -2.1 | ns | ns |
| Amygdala L | 1.7 | 0.2 | 1.0 | 0.1 | 0.9 | 0.1 | -4.4 | -5.1 | <0.001 | <0.001 |
| Caudate R | 2.2 | 0.2 | 2.0 | 0.2 | 1.9 | 0.2 | -0.1 | -0.5 | ns | ns |
| Caudate L | 2.3 | 0.3 | 1.8 | 0.3 | 1.7 | 0.4 | -1.4 | -1.9 | ns | ns |
| Putamen R | 3.2 | 0.5 | 2.7 | 0.2 | 2.5 | 0.2 | -0.8 | -1.1 | ns | 0.004 |
| Putamen L | 3.3 | 0.5 | 2.2 | 0.4 | 2.0 | 0.3 | -1.8 | -2.2 | 0.021 | <0.001 |
| Thalamus R | 6.0 | 0.6 | 6.1 | 0.6 | 6.0 | 0.5 | 0.7 | 0.4 | ns | ns |
| Thalamus L | 6.3 | 0.7 | 5.8 | 0.6 | 5.7 | 0.6 | -0.3 | -0.5 | ns | ns |

This table shows the differences in SOI volumes (ml) at baseline between 6 patients with svPPA at baseline and at one-year follow up versus controls (n=39). Mean and standard deviation are reported for each group. Z-scores were calculated as the difference between the subject's mean and the control group's mean, divided by the control group's standard deviation. FDR-corrected *p* values are reported if significant.

**Legend**: SOIs: Structures of Interest; ABV: Atlas-based-Volumetry;svPPA: semantic variant of primary progressive aphasia; FU: follow-up; SD: standard deviation; GM: gray matter; WM: white matter; ns: non significant.

**Supplementary Table 7**

| **Estimators** | **Max Depth** | **Fold 1 Accuracy** | **Fold 2 Accuracy** | **Fold 3 Accuracy** | |  | **Fold 4 Accuracy** | | | **Fold 5 Accuracy** | **Averaged Cross Validated Accuracy** | **Standard Deviation Cross Validated Accuracy** |
| --- | --- | --- | --- | --- | --- | --- | --- | --- | --- | --- | --- | --- |
| 100 | 4 | 0.91 | 0.91 | | 0.64 | | | 0.82 |  | 0.73 | 0.80 | 0.11 |

This table shows accuracy values for each fold of a 5-fold cross-validation procedure performed using a Random Forest classifier. The classifier was implemented using 100 decision trees and the Gini index as the splitting criterion. The maximum depth of the trees was set to 4, and feature subsets were selected using the square root of the total number of features at each split (. The last two columns report the average cross-validated accuracy and its standard deviation across the five folds.

**Supplementary Table 8** Cross sectional differences in the SOIs between healthy controls and participants with subjective cognitive decline

| **ABV analysis** | **Healty Controls** | | **Subjective Cognitive Decline** | | **Group Comparison** |
| --- | --- | --- | --- | --- | --- |
|  | Mean | SD | Mean | SD | svPPA vs nfPPA |
| Cerebrum GM | 501.0 | 65.7 | 542.9 | 48.0 | ns |
| Cerebrum WM | 393.2 | 20.8 | 390.8 | 33.4 | ns |
| Frontal lobe R | 163.0 | 22.1 | 175.5 | 16.9 | ns |
| Frontal lobe L | 130.6 | 9.1 | 132.7 | 12.3 | ns |
| Temporal lobe R | 146.9 | 12.7 | 153.0 | 9.8 | ns |
| Temporal lobe L | 146.8 | 13.3 | 155.2 | 9.7 | ns |
| Parietal lobe R | 90.0 | 7.6 | 93.0 | 5.4 | ns |
| Parietal lobe L | 90.7 | 6.9 | 94.8 | 6.3 | ns |
| Occipital lobe R | 80.4 | 6.7 | 83.9 | 4.7 | ns |
| Occipital lobe L | 82.3 | 7.2 | 87.6 | 5.3 | ns |
| Insula R | 60.1 | 4.9 | 62.9 | 4.4 | ns |
| Insula L | 57.4 | 5.7 | 61.0 | 4.4 | ns |
| Cerebellum | 7.9 | 0.9 | 8.1 | 0.7 | ns |
| Brainstem | 8.1 | 0.9 | 8.5 | 0.6 | ns |
| Hippocampus R | 3.2 | 0.3 | 3.4 | 0.3 | ns |
| Hippocampus L | 3.1 | 0.3 | 3.3 | 0.3 | ns |
| Amygdala R | 1.8 | 0.1 | 1.9 | 0.2 | ns |
| Amygdala L | 1.7 | 0.1 | 1.8 | 0.2 | ns |
| Caudate R | 2.1 | 0.3 | 2.2 | 0.3 | ns |
| Caudate L | 2.2 | 0.3 | 2.3 | 0.3 | ns |
| Putamen R | 2.9 | 0.4 | 3.3 | 0.5 | ns |
| Putamen L | 2.9 | 0.4 | 3.4 | 0.5 | ns |
| Thalamus R | 5.8 | 0.6 | 6.1 | 0.6 | ns |
| Thalamus L | 6.0 | 0.7 | 6.4 | 0.6 | ns |

This table shows the differences in SOI volumes (ml) at baseline between 10 healthy controls and 19 participants with subjective cognitive decline. Mean and standard deviation are reported for each group. FDR-corrected *p* values are reported if significant.

**Supplementary Table 9** Cross sectional differences in the TOIs between healthy controls and participants with subjective cognitive decline

| **Tract of interest (TOI) analysis** | **Healthy controls (HC)** | | **Participants with subjective cognitive decline (SCD)** | | **HC vs SCD (p value )** |
| --- | --- | --- | --- | --- | --- |
|  | **Mean** | **SD** | **Mean** | **SD** |  |
| Left uncinate fasciculus | 0.30 | 0.02 | 0.29 | 0.02 | ns |
| Right uncinate fasciculus | 0.29 | 0.03 | 0.27 | 0.02 | ns |
| Genu of the corpus callosum | 0.37 | 0.05 | 0.33 | 0.05 | ns |
| Splenium of the corpus callosum | 0.37 | 0.05 | 0.36 | 0.05 | ns |
| Section II of the corpus callosum | 0.28 | 0.02 | 0.28 | 0.02 | ns |
| Section III of the corpus callosum | 0.31 | 0.05 | 0.30 | 0.03 | ns |
| Section IV of the corpus callosum | 0.37 | 0.07 | 0.35 | 0.03 | ns |
| Left superior longitudinal fasciculus | 0.35 | 0.04 | 0.35 | 0.02 | ns |
| Right superior longitudinal fasciculus | 0.39 | 0.04 | 0.38 | 0.03 | ns |
| Left inferior longitudinal fasciculus | 0.32 | 0.03 | 0.31 | 0.03 | ns |
| Right inferiori longitudinal fasciculus | 0.33 | 0.04 | 0.31 | 0.03 | ns |
| Inferior fronto-occipitalis fasciculus | 0.30 | 0.03 | 0.30 | 0.02 | ns |
| Cingulum | 0.39 | 0.05 | 0.38 | 0.03 | ns |
| Pontine projections | 0.37 | 0.03 | 0.35 | 0.02 | ns |
| Anterior thalamic radiation | 0.29 | 0.03 | 0.28 | 0.02 | ns |
| Corticostriatal projections | 0.29 | 0.03 | 0.29 | 0.02 | ns |
| Corticospinal tract | 0.38 | 0.03 | 0.37 | 0.03 | ns |
| Optic radiation | 0.37 | 0.04 | 0.37 | 0.03 | ns |
| Fornix | 0.27 | 0.04 | 0.27 | 0.02 | ns |
| Left tapetum | 0.34 | 0.06 | 0.33 | 0.02 | ns |
| Right tapetum | 0.38 | 0.05 | 0.37 | 0.03 | ns |

This table shows the differences in mean fractional anisotropy (FA) values at baseline between 10 healthy controls and 29 participants with subjective cognitive decline. No significant difference could be detected after correction for multiple comparison. Additionaly. mean and standard deviation are reported for each TOI for documentation purposes.

**Legend** TOIs: Tracts of Interest; SD: Standard Deviation.

**Supplementary Figure 1** Whole brain differences in Fractional Anistropy between subjects with Subjective Cognitive Decline and Healthy Controls


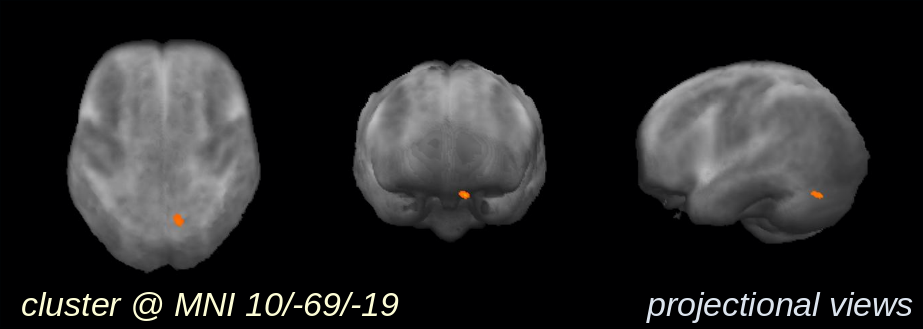


WBSS of 10 Healthy Controls vs 29 subjects with subjective cognitive decline revealed only one small cluster of increase in the cerebellum after correction for multiple comparisons (size 277 mm³. MNI 10/-69/-19). which disappeared after spatial correction.

**Legend**: WBSS: Whole Brain Spatial Statistics
